# A molecular roadmap of the AGM region reveals BMPER as a novel regulator of HSC maturation

**DOI:** 10.1084/jem.20162012

**Published:** 2017-12-04

**Authors:** Alison C. McGarvey, Stanislav Rybtsov, Céline Souilhol, Sara Tamagno, Ritva Rice, David Hills, Duncan Godwin, David Rice, Simon R. Tomlinson, Alexander Medvinsky

**Affiliations:** 1 Stem Cell Bioinformatics Group, Institute for Stem Cell Research, Medical Research Council Centre for Regenerative Medicine, University of Edinburgh, Edinburgh, Scotland, UK; 2 Ontogeny of Haematopoietic Stem Cells Group, Institute for Stem Cell Research, Medical Research Council Centre for Regenerative Medicine, University of Edinburgh, Edinburgh, Scotland, UK; 3 University of Helsinki and Helsinki University Hospital, Helsinki, Finland

## Abstract

In the developing embryo, hematopoietic stem cells (HSCs) emerge from the aorta-gonad-mesonephros (AGM) region, but the molecular regulation of this process is poorly understood. Recently, the progression from E9.5 to E10.5 and polarity along the dorso-ventral axis have been identified as clear demarcations of the supportive HSC niche. To identify novel secreted regulators of HSC maturation, we performed RNA sequencing over these spatiotemporal transitions in the AGM region and supportive OP9 cell line. Screening several proteins through an ex vivo reaggregate culture system, we identify BMPER as a novel positive regulator of HSC development. We demonstrate that BMPER is associated with BMP signaling inhibition, but is transcriptionally induced by BMP4, suggesting that BMPER contributes to the precise control of BMP activity within the AGM region, enabling the maturation of HSCs within a BMP-negative environment. These findings and the availability of our transcriptional data through an accessible interface should provide insight into the maintenance and potential derivation of HSCs in culture.

## Abstract (precis)

Through transcriptional profiling of the mouse AGM region, McGarvey et al. identify potential niche regulators of HSC development. They show a new function of BMPER in regulating HSC maturation, likely via its modulation of BMP signalling.

## Introduction

Hematopoietic stem cells (HSCs), defined by their capacity to provide long-term reconstitution of the entire blood system, first appear in a region of mammalian embryos called the aorta-gonad-mesonephros (AGM) region (Medvinsky et al., 1993; Medvinsky and Dzierzak, 1996). The highly potent nature of these cells makes them of interest in hematological disease studies as well as being one of the key paradigms of tissue maintenance and regeneration by stem cells. Elucidating the processes governing the formation of HSCs from their embryonic precursors not only gives insight into how a stem cell system is established in the embryo but also informs the potential generation of HSCs in vitro for clinical use.

In the mouse, transplantable HSCs in the AGM region can first be detected between E10.5 and E11.5 (Müller et al., 1994; Medvinsky and Dzierzak, 1996; Kumaravelu et al., 2002) and are preceded by the appearance of adult-type spleen colony forming progenitors (CFU-S) at embryonic day (E) 9.5 (Medvinsky et al., 1993). The developmental origins of HSCs are closely associated with endothelial cells. Indeed, the coexpression of early hematopoietic (Runx1, Sca1, Kit, CD34) and endothelial (VE-cadherin [VC], CD31) markers in the dorsal aorta endothelium and intraluminal clusters of cells attached to this endothelium suggests an endothelial origin of HSCs (Jaffredo et al., 1998; de Bruijn et al., 2002; North et al., 2002; Taoudi et al., 2005; Chen et al., 2009; Boisset et al., 2010; Zovein et al., 2010; Guiu et al., 2013; Yvernogeau and Robin, 2017). The development of a reaggregate ex vivo culture system has enabled the origins of HSCs to be directly traced back to a series of precursor populations (pro/preHSCs) as early as E9.5 (Taoudi et al., 2008; Rybtsov et al., 2011, 2014). These precursors express VC, indicative of their endothelial origin, and sequentially up-regulate the hematopoietic markers CD41, CD43, and CD45 during their development (Taoudi et al., 2008; Rybtsov et al., 2011, 2014).

The lack of a repopulating potential of HSC precursor cells indicates a priori that these cells require specific extrinsic cues to reach a mature HSC state. This maturation process can, with some degree of efficiency, occur upon transplantation into a newborn environment (Yoder and Hiatt, 1997). This process can be recapitulated more controllably and robustly ex vivo in AGM explants (Medvinsky and Dzierzak, 1996; de Bruijn et al., 2000; Cumano et al., 2001; Taoudi and Medvinsky, 2007), in reaggregates with AGM stromal cells (Taoudi et al., 2008), in coaggregates with OP9 (stromal cell line derived from calvaria of newborn osteopetrotic [op/op] mice) stromal cells as a surrogate minimal niche (Rybtsov et al., 2011), or in recent modifications of this system (Hadland et al., 2015; Zhou et al., 2016). The signals emanating from the embryonic HSC niche are therefore key to understanding HSC development and ultimately to directing differentiation of pluripotent cells to transplantable HSCs in vitro*.*

A clear path toward identifying the HSC inductive signals is to learn from the in vivo process. Recent in vitro modeling of HSC development has revealed that the AGM region exhibits a dorsal-ventral polarity, with HSCs emerging predominantly from the ventral region (Taoudi and Medvinsky, 2007; Souilhol et al., 2016a). Although the ventral domain of the E10.5 dorsal aorta (AoV) provides an immediate supportive environment for HSC generation, the efficiency of this process is enhanced by the dorsal domain of the dorsal aorta (AoD) and urogenital ridges (UGRs), indicating a high complexity of the niche likely formed by long- and short-range cross signaling (Souilhol et al., 2016a). Additionally, although a 4-d E10.5 AGM culture is sufficient to support HSC formation, HSC development from E9.5 caudal part requires a 7-d culture, and even then, supplement with cytokines and OP9 cells is necessary (Rybtsov et al., 2014). A dramatic expansion of pro/preHSC numbers from E9.5 (1–2 HSCs) to E10.5 (50 HSCs; Rybtsov et al., 2016) along with gradual pro/preHSC maturation suggests significant transitions of the supportive niche within this developmental window. Therefore, here we have taken the dorso-ventral polarity and the E9.5 to E10.5 niche transition as guides for analysis of the environment supporting HSC development.

Whole-transcriptome expression profiling, and particularly next-generation sequencing technologies, are now powerful tools for molecular characterization. Several studies, largely focused on enriched populations of developing hematopoietic progenitors and HSCs, have implemented these technologies to gain important insights (McKinney-Freeman et al., 2012; Swiers et al., 2013; Li et al., 2014; Solaimani Kartalaei et al., 2015). Studies of the embryonic HSC niche so far have focused on stromal cell lines (Charbord et al., 2014) or have lacked the spatial resolution along the dorsal-ventral axis (Mascarenhas et al., 2009). The validation of these profiles is hampered by the reliance on very low throughput methods for functional screening such as mouse knockouts, or testing in distant species. Here we sought to elucidate a more complete model of the inductive signaling coming from the mouse HSC niche through transcriptional profiling of the spatiotemporal transitions in the AGM region and functional validation of our findings with a tractable reaggregate culture system.

Given our understanding of the spatial and temporal demarcation of the AGM region’s functionality, we dissected AGM domains with differing HSC supportive potentials and through RNA sequencing (RNA-seq) identified their key molecular signatures. In addition, we have characterized an in vitro supportive environment, OP9 cells, by RNA-seq and compared these profiles. Coupling this analysis with reaggregate culture and transplantations has enabled us to explore the role of several secreted molecules expressed during developmental progression of the AGM region. We have identified *Bmper*, a ventrally polarized gene whose expression increases between E9.5 and E10.5, as a novel regulator of HSC development in the AGM region. To facilitate further studies of molecular regulators of HSC development, we present our molecular roadmap of the developing HSC niche through a dynamic interface (http://agmniche.stembio.org/) to enable easy mining of this data resource.

## Results

### Transcriptome profiling of the spatial and temporal transitions in the in vivo HSC niche

**Figure 1.**
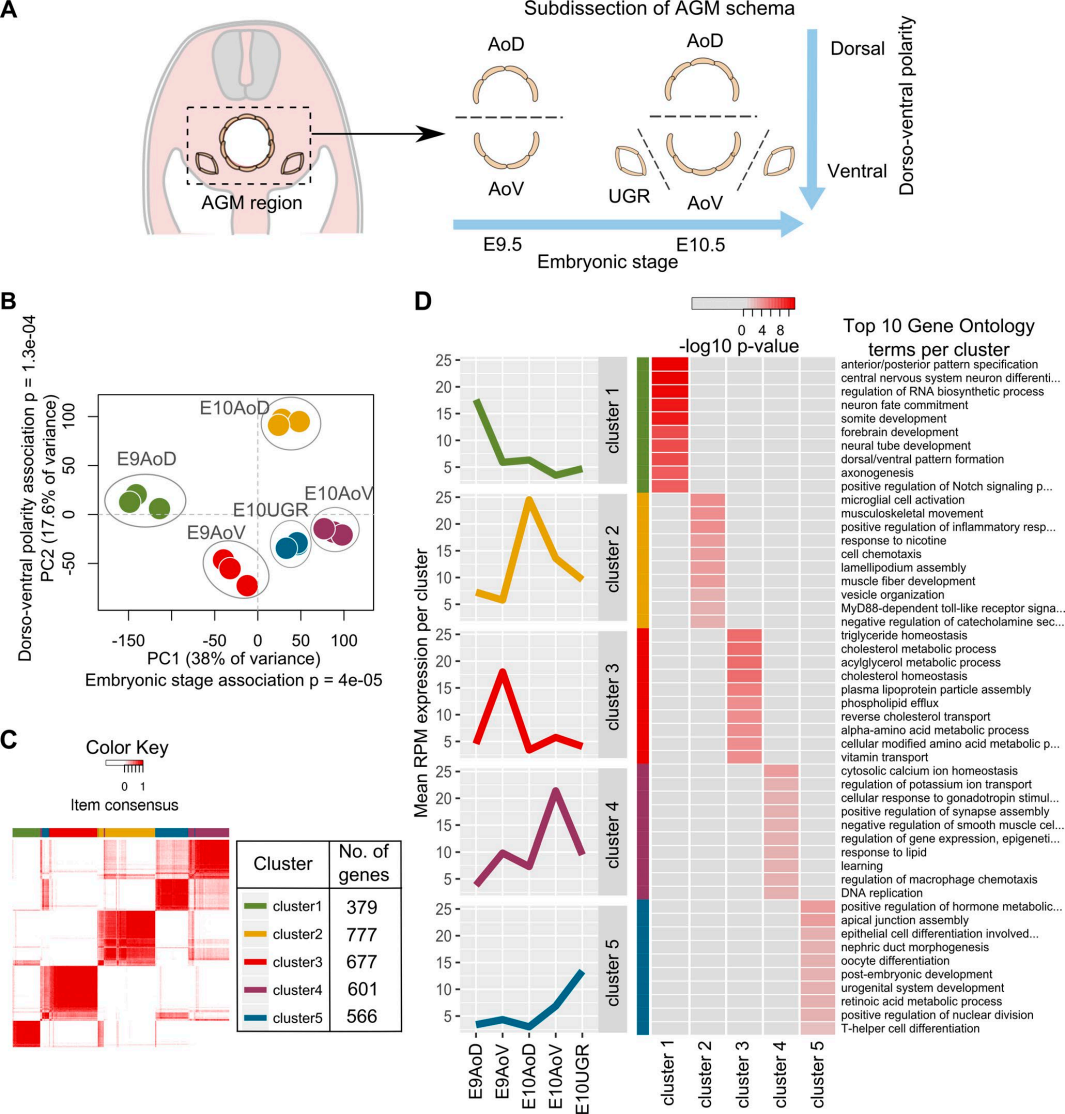
**Transcriptional profiling and identification of unique gene signatures underlying differing functionality of AGM subdomains.** (A) The AGM from E10.5 and E9.5 embryos, subdissected into AoD, AoV, and UGR for RNA-seq in triplicate. (B) AGM samples plotted based on the first two principal components of all normalized expression values. Significant association of stage and dorsal-ventral position with the first two principal components tested by ANOVA. (C) The 3,000 most variant genes across all AGM samples divided into five stable gene clusters with ConsensusCluster. Clusters are depicted by a consensus matrix heat map where each row and column represents a unique gene and the color scale represents consensus values, which are the proportion of times that two items occupied the same cluster out of 50 iterations. The color key indicates cluster definitions and the number of genes in each cluster. (D) The expression profile of each gene cluster mean and top 10 gene ontology terms enriched in each cluster. Heat map colors represent p-values from a weighted Fisher’s test transformed by −log10.

The AGM region acquires the capacity to autonomously support HSC development between E9.5 and E10.5 in a ventrally polarized manner (Taoudi and Medvinsky, 2007; Rybtsov et al., 2014, 2016; Souilhol et al., 2016a). To capture the molecular basis of these spatial and temporal transitions, we subdissected the E9.5 and E10.5 AGM regions into the dorsal and ventral parts (E9 AoD, E9 AoV, E10 AoD, and E10 AoV), and at E10.5 additionally separated the urogenital ridges (E10 UGR; Fig. 1 A). By pooling dissected tissues from between 15 and 34 embryos in three separate experiments, we yielded sufficient RNA to sequence genome-wide without the need for preamplification.

To assess the overall similarities in expression profiles between subdomains, we plotted the samples based on their principal components (Fig. 1 B). The first two principal components, which represent 38% and 17.6% of the variance in the data, respectively, group the samples into distinct clusters based on their tissue of origin. Indeed, an ANOVA indicates that PC1 significantly partitions samples by embryonic stage (P = 4 × 10^−5^) and PC2 by dorsal-ventral polarity (P = 1.3 × 10^−4^; Table S1). Hence, the AGM subdomains show clear differences based on their expression profiles, and the primary variance in these profiles is attributed to our spatial and temporal transitions of interest.

### Unsupervised clustering analysis elucidates the molecular signatures of AGM region tissues

To identify groups of genes that show similar patterns of expression dynamics across the samples, we applied an unsupervised genewise clustering method. The 3000 most dynamically expressed genes were grouped into clusters based on a correlation of each gene’s expression profile across the samples. The most stable grouping consisted of five clusters of genes (Fig. 1 C, Fig. S1, and Table S2) varying in size from 379 to 777 genes, which show distinct gene expression profiles.

After identifying five distinct gene clusters by this unsupervised method, we assessed their expression profiles relative to the sample phenotypes. We found that the mean expression profiles of each cluster show distinct peaks that each correspond to different AGM subdomains, thus representing their specific molecular signature (Fig. 1 D and Fig. S1 D). Specifically, E9 AoD is characterized by cluster 1, E10 AoD by cluster 2, E9 AoV by cluster 3, E10 AoV by cluster 4, and E10 UGR by cluster 5. This association between gene clusters and tissue phenotypes is supported by the significant enrichment of gene ontologies in these gene clusters, which generally reflect the known anatomy of the AGM region. For example, cluster 1 (E9 AoD) is enriched for gene ontologies associated with dorsal tissues such as “somite development” and “neural tube development,” whereas cluster 5 (E10 UGR) is enriched for the terms “nephric duct formation,” “oocyte differentiation,” and “urogenital system development.” Both gene clusters associated with E10.5 dorsal aorta (cluster 2, E10 AoD; and cluster 4, E10 AoV) are enriched for hematopoietic ontologies, in agreement with the high hematopoietic activity at this stage and location. The partial overlap of genes in clusters 4 and 5 (Fig. 1 C) agrees with the proximity of E10 AoV and E10 UGR samples seen by principal component analysis (Fig. 1 B), likely because of their anatomical vicinity.

By elucidating molecular profiles representing each of the AGM domains, we generated a data resource that enables exploration of key short- and long-range signaling acting on HSC precursors as they mature. To enable simple data exploration, we have constructed a dynamic visualization model to enable users to select tissues of interest, filter by expression level and molecule type, and select precomputed clusters. We have further linked this model to existing databases, the Mouse Atlas Project (Richardson et al., 2014) and Ensembl (Yates et al., 2016), to enable users to gather reference data and validate their filtering (http://agmniche.stembio.org/).

### Pathway enrichment analysis reveals the dynamic signaling activity in the AGM niche

**Figure 2.**
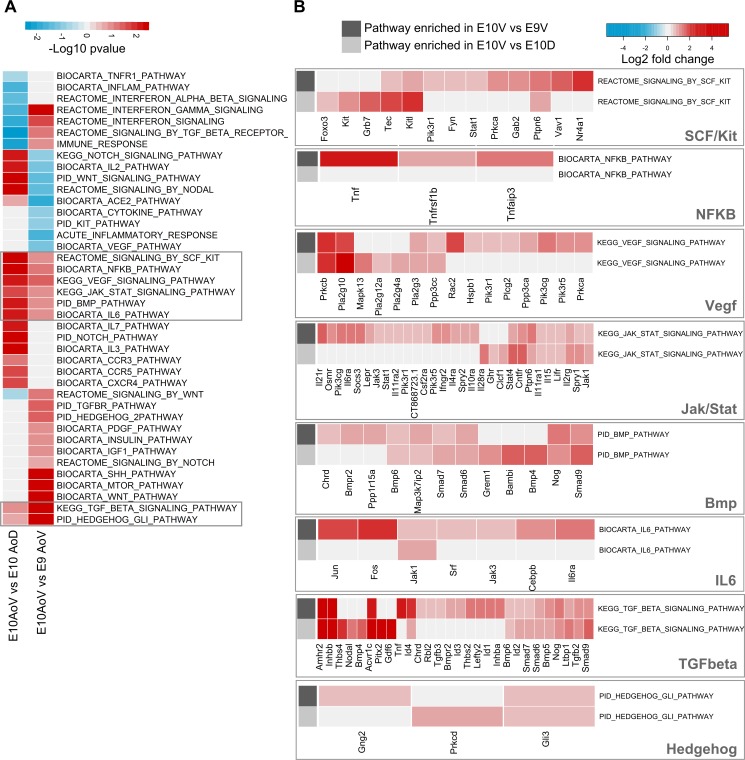
**Pathway enrichment during spatial and temporal transitions of the HSC niche.** (A) Differential gene set testing of hematopoiesis-associated canonical pathways from the Molecular Signatures Database between E10 AoV versus E9 AoV or E10 AoV versus E10 AoD calculated by Limma ROAST. Color scale represents −log10 multiple hypothesis corrected p-values. The pathways significantly enriched in both comparisons are outlined in gray. (B) Genes contributing to the enrichment of each of the signaling pathways up-regulated in both E10 AoV versus E9 AoV and E10 AoV versus E10 AoD. Heat map color indicates either the fold change expression in E10.5 versus E9.5 (rows indicated by dark gray) or fold change expression in E10 AoV versus E10 AoD (rows indicated by light gray).

We focused in more detail on the genes and pathways uniquely expressed in E10.5 AoV. From the ontologies enriched in cluster 4, we observed “positive regulation of angiogenesis” and “blood vessel endothelial cell migration” (Table 1) possibly linked to the endothelial remodeling required for intra-aortic cluster formation (Zovein et al., 2010) as well as “stem cell maintenance” and “male gonad development,” consistent with the migration of primordial germ cells through the ventral mesenchyme at E10.5 en route to the urogenital ridges (Medvinsky et al., 1996; Molyneaux et al., 2001; Yokomizo and Dzierzak, 2010). We also saw enrichment for “regulation of BMP signalling pathway,” as well as the proinflammatory signatures “regulation of macrophage chemotaxis” and “regulation of interferon-gamma production,” both of which have recently been implicated in regulation of HSC emergence (Wilkinson et al., 2009; Espín-Palazón et al., 2014; Li et al., 2014; Pouget et al., 2014; Sawamiphak et al., 2014; Crisan et al., 2015; Souilhol et al., 2016a). Differential gene set testing of pathways associated with the regulation of HSC development also identified a significant expression of BMP signaling and proinflammatory pathways NF-κB, Jak/Stat, and IL-6 in E10 AoV relative to E9 AoV and E10 AoD (Fig. 2, A and B). SCF/Kit, VEGF, and TGF-β were also enriched in E10 AoV (Fig. 2 B), consistent with their previously described roles in HSC maturation and survival (Gering and Patient, 2005; Bowie et al., 2007; Taoudi et al., 2008; Ciau-Uitz et al., 2013; Rybtsov et al., 2014; Monteiro et al., 2016; Souilhol et al., 2016a). Furthermore, hedgehog signaling downstream of Gli was enriched in AoV, suggesting activation of this pathway is important for HSC formation by dorsally polarized Shh (Gering and Patient, 2005; Peeters et al., 2009; Souilhol et al., 2016a).

**Table 1.** Gene ontology enrichment in cluster 4

GO.ID	Term	Annotated	P-value (weight Fisher’s)	Genes contributing to enrichment
GO:0010758	Regulation of macrophage chemotaxis	7	0.00355	*C5ar1*, *Ccl2*, *Mmp28*, *Ptk2b*, *Rarres2*
GO:0098542	Defense response to other organism	62	0.00776	*C5ar1*, *Cxcl1*, *Ddx60*, *Epx*, *Fgr*, *Gbp2*, *Iigp1*, *Il27ra*, *Irf5*, *Isg15*, *Lyz1*, *Mpo*, *Mx2*, *Naip6*, *Nlrc4*, *Oasl2*, *Oprk1*, *Spn*, *Spon2*, *Syk*
GO:0032649	Regulation of IFN-γ production	10	0.02583	*Fzd5*, *Il27ra*, *Isg15*, *Isl1*, *Runx1*
GO:0060638	Mesenchymal-epithelial cell signaling	5	0.00537	*Fgf10*, *Hgf*, *Tnc*, *Wnt2b*
GO:0001706	Endoderm formation	14	0.00829	*Dusp1*, *Gata4*, *Gata6*, *Inhba*, *Nanog*, *Nog*, *Pou5f1*
GO:0045766	Positive regulation of angiogenesis	32	0.01032	*Angpt2*, *C5ar1*, *Ccbe1*, *Ctsh*, *Gata4*, *Gata6*, *Hgf*, *Isl1*, *Prkcb*, *Ptk2b*, *Runx1*, *Srpx2*
GO:0043534	Blood vessel endothelial cell migration	13	0.02281	*Angpt2*, *Egr3*, *Klf4*, *Nr4a1*, *Ptk2b*, *Srpx2*
GO:0030510	Regulation of BMP signaling pathway	30	0.01664	*Bmper*, *Cav1*, *Chrdl2*, *Crb2*, *Fstl3*, *Gata4*, *Gata6*, *Gdf3*, *Kcp*, *Nanog*, *Nog*
GO:0071371	Cellular response to gonadotropin stimulation	9	0.00222	*Egr2*, *Egr3*, *Egr4*, *Gata4*, *Gata6*, *Inhba*
GO:0019827	Stem cell maintenance	30	0.01664	*Dppa2*, *Esrrb*, *Fgf10*, *Klf4*, *Nanog*, *Nog*, *Phf19*, *Piwil2*, *Pou5f1*, *Prdm14*, *Tcl1*
GO:0008584	Male gonad development	26	0.02506	*Esr2*, *Fgf9*, *Gata4*, *Inhba*, *Lhx9*, *Nupr1*, *Rxfp2*, *Tcf21*, *Tex19.1*, *Zfpm2*

Selected gene ontologies that are significantly enriched in cluster 4 by weighted Fisher's test (P < 0.05) and the genes that contribute to this enrichment.

Within the AGM region, *Bmp4* expression is spatially polarized to the AoV (Marshall et al., 2000; Durand et al., 2007; Wilkinson et al., 2009; Crisan et al., 2015; Souilhol et al., 2016a). Our analysis identified a range of additional BMP/TGF-β ligands such as *Bmp5*, *Bmp6*, *Inhbb*, *Inhba*, and *Tgfb2* preferentially expressed in E10 AoV, which could influence HSC development (Table 1 and Fig. 2). However, we also see the enrichment of several inhibitory and regulatory molecules such as *Nog*, *Grem1*, *Chrd*, *Bmper*, *Chrdl2*, *Fstl3*, *Kcp* and, importantly, inhibitory *Smad6* and *Smad7* (Table 1 and Fig. 2), some of which are also observed ventrally in zebrafish aorta (Wilkinson et al., 2009). This reveals a significant negative BMP signaling component in the E10 AoV niche, in keeping with the recent finding that BMP4 inhibition is necessary for HSC maturation within the AGM region (Souilhol et al., 2016a).

The consistent enrichment of proinflammatory signaling pathways in E10 AoV such as NF-κB, Jak/Stat, and IL-6 (Fig. 2) along with the enrichment for GO terms “macrophage chemotaxis” and “regulation of interferon-gamma production” (Fig. 1 D), suggest that this is a key signaling pathway in the developing HSC environment. Given the positive role of IFN-γ in embryonic HSC formation in mouse and zebrafish (Li et al., 2014; Sawamiphak et al., 2014), and TNF in zebrafish (Espín-Palazón et al., 2014), we considered that further proinflammatory cytokines may be important regulators of preHSC maturation in the AGM region.

### Comparative transcriptome analysis reveals a common molecular signature between the in vivo AoV and in vitro OP9 niches

**Figure 3.**
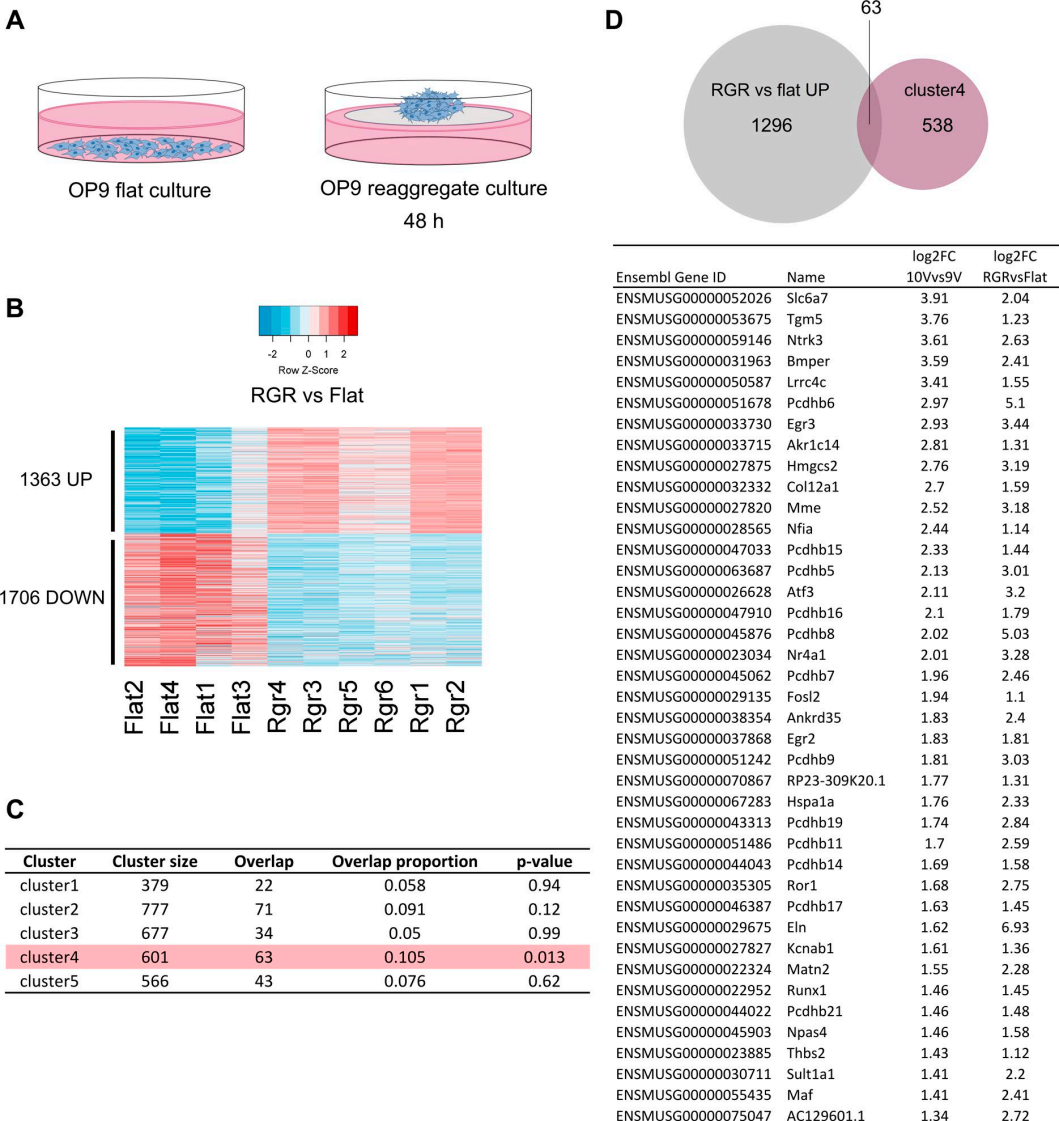
**Comparative analysis reveals common in vivo and in vitro signature for support of HSC maturation.** (A) Transcriptomes of OP9 cells cultured on plastic, submersed in media (left) and cultured on a membrane at the air liquid interface for 48 h after reaggregation (right) were captured by RNA-seq. (B) Differentially expressed genes in reaggregate versus flat culture represented in a heat map showing 1,363 significantly up- and 1,706 significantly down-regulated genes, absolute fold change ≥2 and FDR ≤0.05. (C) Comparison of genes of each AGM gene cluster, with the 1,363 genes up-regulated in OP9 cells after reaggregation. Row highlighted in pink indicates a significant overlap, with p-value <0.05 from hypergeometric test. (D) Top 40 genes in the overlap between AGM cluster 4 (E10 AoV) and the 1,363 genes up-regulated in OP9 after reaggregation, ranked by differential expression in E10 AoV versus E9 AoV.

The OP9 cell line, derived from calvaria of newborn op/op mice (Nakano et al., 1994; Kobayashi et al., 1996), is capable of supporting HSC maturation in vitro from precursors as early as E9.5 in coaggregates with SCF, FLT3L, and IL-3 (Rybtsov et al., 2011, 2014). Moreover, the enforced aggregation and culture at the air-liquid interface on a floating membrane has been shown to enhance HSC maturation compared with flat culture submersed in media (Taoudi et al., 2008). To elucidate the differences between these culture conditions and compare the OP9 expression profile with AGM tissues, we sequenced the transcriptomes of OP9 cells in flat submersed conditions, and after reaggregation followed by culture on a membrane for 48 h (Fig. 3 A).

We observed a significant transcriptional change in OP9 cells that had been cultured in reaggregate compared with submersed conditions, with 1363 up-regulated genes and 1706 down-regulated genes (Fig. 3 B) including integrin, Notch and cell cycle pathways (Table S4). Given the supportive nature of OP9 cells for HSC maturation in the reaggregate culture system (Rybtsov et al., 2011, 2014), we compared the OP9 expression profile to our in vivo gene expression profiles. The 1363 significantly up-regulated genes after reaggregation significantly overlapped only with gene cluster4 suggesting a close resemblance with the supportive in vivo E10.5 AoV environment (Fig. 3 C). The common molecular program between OP9 and E10.5 AoV (Fig. 3 D and Table S5) includes the key hematopoietic transcriptional regulator *Runx1*; several other molecules associated with lymphoid regulation, *Mme* (acute lymphocytic leukemia antigen), *Egr2*, and *Egr3* (Li et al., 2012); several extracellular structural proteins, *Col12a1*, *Elastin*, *Matn2*, *Thbs2*; signaling molecules associated with neural development, *Ntrk3*, protocadherins, and *Ror1*; and a modulator of BMP signaling, *Bmper*.

### Functional screen identifies BMPER as HSC development regulator

**Figure 4.**
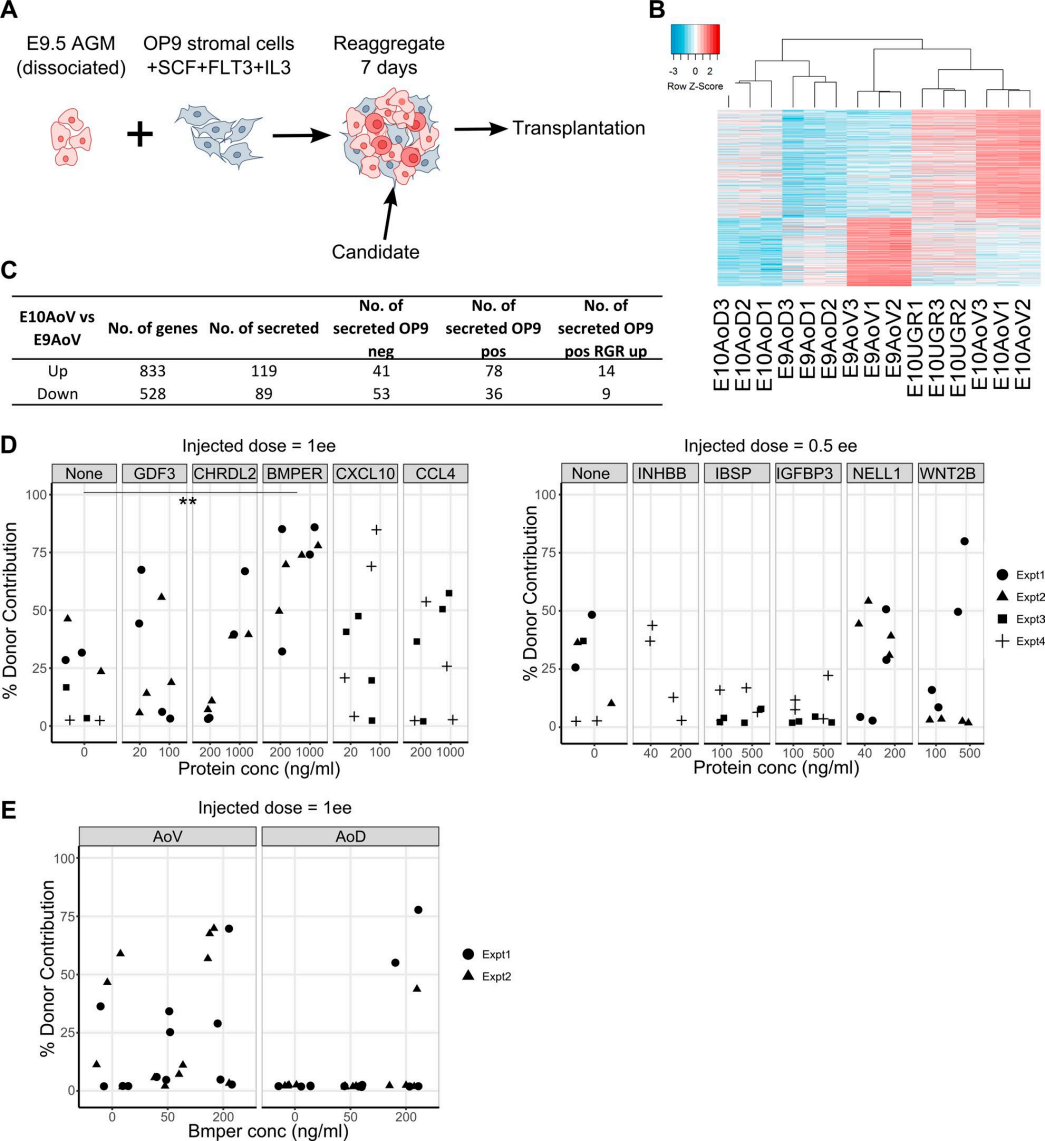
**Screening secreted factors expressed highly in E10.5 AoV identifies a functional role of BMPER on HSC maturation.** (A) Experimental setup to test candidates from the bioinformatics analysis. 7-d culture of E9.5 caudal part that was dissociated and reaggregated with OP9 cells, SCF, IL-3, and FLT3L, followed by transplantation. (B) Differentially expressed genes in E10 AoV versus E9 AoV represented in the heat map showing 833 significantly up- and 528 significantly down-regulated genes with absolute fold change ≥2 and FDR ≤0.05. (C) Table quantifies the numbers of differentially expressed genes in E10 AoV versus E9 AoV; differentially expressed secreted factors (gene ontology GO:0005576 extracellular region or GO:0005615 extracellular space); and not expressed in OP9, expressed in OP9, or expressed significantly higher in OP9 upon reaggregation (expression threshold >0.5 RPM). (D) Percentage contribution of donor cells 16 wk after injection with E9.5 caudal part cells cultured with the named recombinant protein at the displayed concentration for 7 d with serum media, OP9 cells and SCF, IL3, and FLT3L (injected dose, 1 e.e. or 0.5 e.e. as indicated). For each condition, experiments were performed at least twice (except INHBB) using pools of different litters of embryos. Points indicate each transplanted mouse, and symbols within graphs indicate batches of reaggregate experiments. Significant increase in repopulation versus control calculated by Wilcoxon rank-sum test and all p-values included in Table S6. **, P = 0.008. (E) Percentage donor contribution 16 wk after injection with E11.5 AoV or AoD cells cultured with BMPER at the displayed concentration for 5 d with in serum-free, cytokine-free media (injected dose, 1 e.e.). For each condition, experiments were performed twice using pools of different litters of embryos. Points indicate each transplanted mouse, and symbols indicate each separate reaggregate experiment.

Compared with E10.5 AoV, maturation of proHSCs from the E9.5 AGM region occurs more slowly and less efficiently in culture (Rybtsov et al., 2014). We hypothesized that the molecules of the E10.5 AoV niche may be supplemented to the E9.5 reaggregate culture and improve the efficiency of HSC production ex vivo (Fig. 4 A). Hence, we focused on genes most up-regulated between E9.5 and E10.5 (Fig. 4 B) that are functionally annotated as secreted. Of 833 significantly up-regulated genes, 119 were secreted (Fig. 4 C), and these were ranked based on differential expression between E10.5 versus E9.5 and AoV versus AoD. Further, we identified secreted candidates that are absent from OP9 or expressed in OP9, or whose expression in OP9 increases upon reaggregation (Fig. 4 C). Those common to OP9 and AGM are likely to be relevant as they are present in two independent supportive cell types, whereas those absent from OP9 may provide insight into the molecules absent from E9.5 stroma that can't be supplemented by OP9 cells. We therefore selected 10 candidates that span these three categories to test their effect on HSC formation. Based on enrichment of the pathways described above (Table 1 and Fig. 2), we focused on significantly expressed BMP/TGF/Nodal components, Bmper, Gdf3, Chrdl2, and Inhbb; proinflammatory cytokines, Cxcl10 (IFN-γ–induced protein 10) and Ccl4 (macrophage inflammatory protein-1β); a homologous protein to the immune adhesion regulator OPN (Lund et al., 2009) called Ibsp (Tagliabracci et al., 2012); and effectors of cell growth and differentiation: Igfbp3 (an insulin-like growth factor regulator), Nell1 (an EGF-like repeat containing protein), and Wnt2b (a conserved WNT family member).

For each of these selected differentially expressed candidates, we added their corresponding recombinant protein in two different doses to our standard reaggregate cultures of E9.5 caudal part with OP9 cells, SCF, FLT3L, and IL-3. After seven days of culture, the reaggregates were assessed for repopulation of sublethally irradiated mice. We found that in all independent experiments, 16 wk after injection, BMPER-treated reaggregates repopulated recipients at significantly higher levels than control cultures (P = 0.008), with all 8 recipients repopulated >25% compared with only 3 out of 8 control mice repopulated >25% (Fig. 4 D and Table S6). Moreover, we observed a dose-dependent effect: 4 of 4 reaggregates cultured with 1 µg/ml BMPER gave >70% contribution compared with a mean of 57% contribution from 200 ng/ml BMPER treated and 17% contribution from controls while showing normal lineage contribution (Fig. S2 A). At the same time, BMPER had no significant effect on the number of committed myeloid progenitors generated in culture, as assessed in methylcellulose culture (Fig. S2 B), indicating that it had a specific role in the maturation of HSCs. A second recombinant protein that showed a noticeable outcome was CXCL10; however, its effect was not highly consistent (P = 0.07). No other recombinant protein treatment gave a statistically significant increase in repopulation versus control (Table S6).

Addition of BMPER to the culture of the more developmentally advanced E11.5 ventral and dorsal domains of the dorsal aorta (AoV and AoD, respectively) demonstrated a slight tendency to increase HSC maturation, which was more evident with E11.5 AoD. Specifically, treatment of E11.5 AoD with 200 ng/ml of BMPER resulted in repopulation of 3 mice compared with no repopulation (out of 8 mice transplanted per condition) with untreated AoD (Fig. 4 E). Thus, through transcriptional profiling coupled to functional screening of reaggregates, we have been able to identify BMPER as a novel regulator of HSC maturation.

### Perivascular cells and subaortic mesenchyme are the main source of BMPER within the AGM region

**Figure 5.**
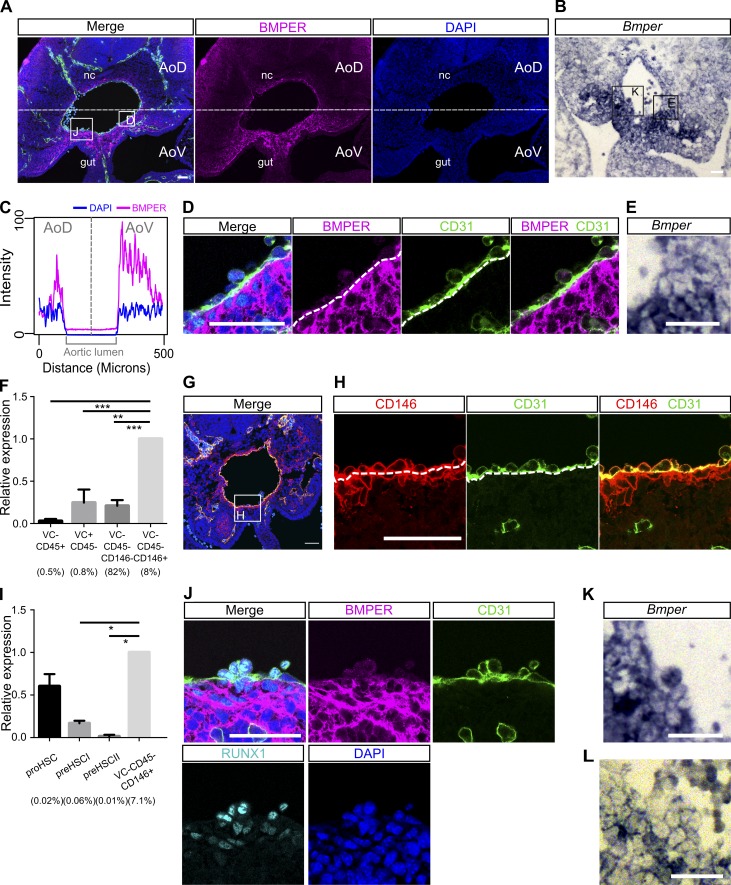
**Perivascular cells and subaortic mesenchyme are the main source of BMPER within the AGM region.** (A) Distribution of BMPER protein in a transverse section of E10.5 AGM measured by immunostaining. Green, CD31; magenta, BMPER; cyan, RUNX1; blue, DAPI. gut, hindgut; nc, notochord. Bar, 50 µm. (B) *Bmper* mRNA in transverse section of the E10.5 AGM region by in situ hybridization. Bar, 50 µm. (C) Quantification of the mean immunostaining signal intensity (mean gray values) of DAPI (blue) and BMPER (magenta) along a box (not depicted) drawn over the dorsal-ventral axis of the AGM region from A. Distance is from the notochord (dorsal), position 0, to the intersection with the gut and the AGM region (ventral), position 500. (D) Higher magnification of the highlighted region from A showing the aortic endothelium and perivascular population. Green, CD31; magenta, BMPER; cyan, RUNX1; blue, DAPI. Bar, 50 µm. (E) Higher magnification of the region highlighted in B showing *Bmper* mRNA around the lining of the aorta. Bar, 50 µm. (F) Expression level of *Bmper* relative to *Tbp* in each sorted population: Lin^−^VC^−^CD45^+^, representing hematopoietic cells; Lin^−^VC^+^CD45^−^, endothelial cells; Lin^−^VC^−^CD45^−^CD146^+^, putative perivascular cells; and Lin^−^VC^−^CD45^−^CD146^−^, remaining stroma. Expression was normalized to the Lin^−^VC^−^CD45^−^CD146^+^ population. Each population as percentage of Lin^−^ cells indicated below. Sorting was performed twice, first from one pool of embryos from four to five litters and second from two pools of embryos from four to five litters. Error bars represent SD from the mean (*n* = 3). Significance calculated by *t* test: **, P = 0.0016; ***, perivascular versus stroma, P = 0.0006; perivascular versus hematopoietic, P = 0.0002. (G) The distribution of nonendothelial, CD146-positive cells and endothelial CD146-positive cells in transverse section of the E10.5 AGM region. Green, CD31; red, CD146; blue, DAPI. Bar, 50 µm. (H) Higher magnification view of the region highlighted in G showing CD31- and CD146-positive endothelial layer and the CD146-positive CD31-negative perivascular layer around the dorsal aorta. Green, CD31; red, CD146; blue, DAPI. Bar, 50 µm. (I) Expression level of *Bmper* relative to *Tbp* in each sorted population: Lin^−^VC^+^CD45^−^CD43^−^CD41^lo^ proHSC, Lin^−^VC^+^CD43^+^CD41^+^ type I preHSC, and Lin^−^VC^+^CD45^+^ type II preHSC and Lin^−^VC^−^CD45^−^CD146^+^ putative perivascular cells. Each population as percentage of Lin^−^ cells indicated below. Sorting was performed twice, from pools of two and six litters, respectively. Expression was normalized to the Lin^−^VC^−^CD45^−^CD146^+^ population. Error bars represent SD from the mean (*n* = 2). Significance calculated by *t* test: *, perivascular versus preHSCI, P = 0.04; perivascular versus preHSCII, P = 0.03. (J) Higher magnification view of highlighted region from A showing the localization of BMPER protein in the hematopoietic clusters of the E10.5 dorsal aorta in sections measured by immunostaining. Magenta, BMPER; green, CD31; cyan, RUNX1; blue, DAPI. Bar, 50 µm. (K and L) Higher magnification view of highlighted region from B (K) and from Fig. S3 C (L) showing *Bmper* mRNA in some but not all cells of the intra-aortic cluster by in situ hybridization. Bars, 50 µm.

Given the functional effect of BMPER on HSC maturation, we investigated the protein and mRNA distribution within the E10.5 AGM niche in more detail. Although not often classified as a canonical BMP pathway member, BMPER (also known as CV-2) has previously been elucidated as a modulator of endothelial cells and a protein capable of binding and modulating BMP signaling (Moser et al., 2003; Kamimura et al., 2004; Rentzsch et al., 2006; Moreno-Miralles et al., 2011; Helbing et al., 2013). Immunostaining for BMPER protein and in situ hybridization against the mRNA in transverse sections of the E10.5 AGM region showed a ventral polarization (Fig. 5, A–C; and Fig. S3), validating our transcriptome data. The protein was observed predominantly in the perivascular layer of cells surrounding the aortic endothelium (Fig. 5, D and E) and extending ventrolaterally into the subaortic mesenchyme (Fig. 5, A–C).

To resolve the expression profile of *Bmper* in greater detail, we sorted E10.5 AGM tissues into key subpopulations: Lin^−^VC^−^CD45^+^, representing hematopoietic cells; Lin^−^VC^+^CD45^−^, endothelial cells; Lin^−^VC^−^CD45^−^CD146^+^, putative perivascular cells; and Lin^−^VC^−^CD45^−^CD146^−^, remaining stroma (Fig. 5 F and Fig. S4 A). By quantitative real-time PCR (qRT-PCR), *Bmper* transcripts were found in endothelial cells, nonendothelial stroma, and with a particularly high degree of enrichment in the Lin^−^VC^−^CD45^−^CD146^+^ population (Fig. 5 F), consistent with the bright immunofluorescence signal and in situ hybridization staining of perivascular (subendothelial) cells (Fig. 5, D, E, G, and H). Mesenchymal (Lin^−^VC^−^CD45^−^CD146^−^) and endothelial (Lin^−^VC^+^CD45^−^) cells express fourfold less *Bmper* than perivascular cells (Fig. 5 F), and the endothelium shows little presence of protein as detected by antibody staining (Fig. 5 D). Thus, although perivascular cells constitute only 8% of cells in the AGM region, given the high expression levels of BMPER and proximity to the dorsal aorta, perivascular cells likely play a key role in BMPER-mediated HSC development.

Little or no expression of *Bmper* was found in VC^−^CD45^+^ hematopoietic cells (Fig. 5 F). To test whether *Bmper* was expressed in the enriched HSC precursor lineage, Lin^−^VC^+^CD45^−^CD43^−^CD41^lo^ proHSC, Lin^−^VC^+^CD45^−^CD43^+^CD41^+^ type I preHSC, and Lin^−^VC^+^CD45^+^ type II preHSC populations were sorted (Taoudi et al., 2008; Rybtsov et al., 2011, 2014) from E10.5 AGM tissues and compared with the Lin^−^VC^−^CD45^−^CD146^+^ population (Fig. 5 I and Fig. S4 B). We found that whereas *Bmper* is expressed in populations of early precursors containing proHSCs, its expression declines in later type I precursors and is practically negligible in type II preHSCs (Fig. 5 I). This is consistent with the relatively low BMPER protein signal in intra-aortic clusters and the presence of *Bmper* mRNA in some but not all cluster cells (Fig. 5, J–L).

Notably, OP9 cells are also CD146 positive, are VC and CD45 negative (Fig. S4, C–E), and express *Bmper*, which is up-regulated after reaggregation (Fig. S4, F–H), further validating the role of BMPER in the supportive developmental HSC niche. This analysis suggests that the supportive characteristics of OP9 cells are a result of similarity in molecular phenotype with in vivo CD146-positive cells in the AoV niche.

### BMPER enhances HSC maturation through precise temporal and spatial modulation of BMP signaling

**Figure 6.**
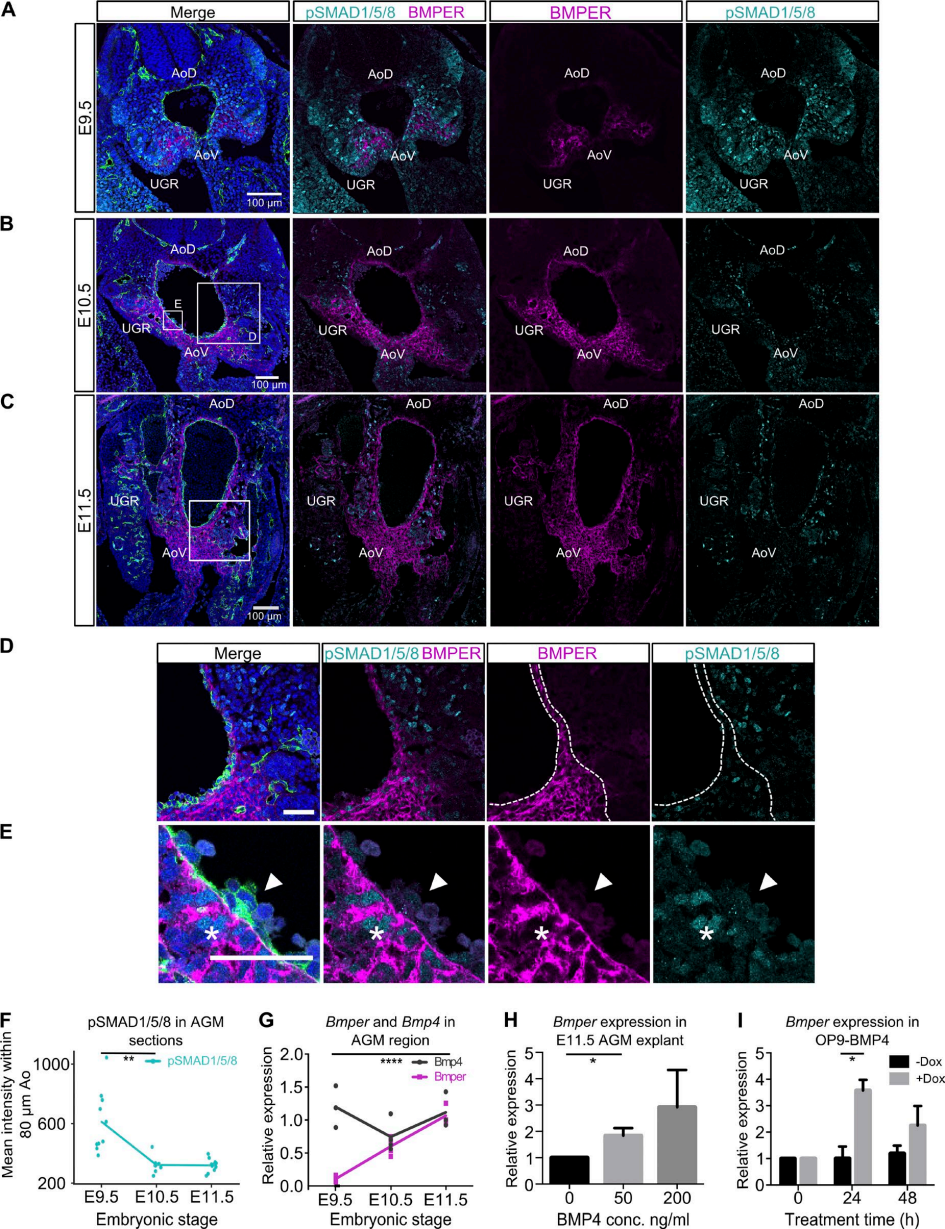
**Negative correlation between distributions of BMPER and pSMAD1/5/8 along with BMP4-induced expression suggests BMPER regulates BMP signaling through negative feedback.** (A–C) Localization of pSMAD1/5/8 and BMPER protein in the AGM region from E9.5 (A), E10.5 (B), and E11.5 (C) embryos. Magenta, BMPER; cyan, pSMAD1/5/8; green, CD31; blue, DAPI. Bars, 100 µm. Boxes highlight regions of complementarity between BMPER and pSMAD1/5/8 shown at higher magnification in D and Fig. S5 A. UGR, one of two indicated. (D) Higher magnification of E10.5 ventro-lateral region highlighted in B. Magenta, BMPER; cyan, pSMAD1/5/8; green, CD31; blue, DAPI. Bar, 50 µm. Dotted line indicates the boundary around regions with high BMPER protein. (E) Higher magnification view of intra-aortic cluster highlighted in box “E” of B. Magenta, BMPER; cyan, pSMAD1/5/8; green, CD31; blue, DAPI. Arrowheads indicate intra-aortic cluster. Asterisks indicate subendothelial cells with strong nuclear pSMAD1/5/8 signal. Bar, 50 µm. (F) Quantification of pSMAD1/5/8 staining intensity (mean gray values) over an 80-µm band around the dorsal aorta on transverse embryo sections from E9.5, E10.5, and E11.5 (shown in Fig. S5 C). Quantification was on sections from at least two embryos (littermates). Significance measured by Student’s *t* test: **, P = 0.0015. (G) Expression of *Bmper* and *Bmp4* in AGMs dissected from E9.5, E10.5, and E11.5 stage embryos normalized to *Tbp*. Each point represents one embryo (littermates). Significance measured by Student’s *t* test: ****, *Bmper* E9.5 versus E11.5 P = 8 × 10^5^. (H) Expression of *Bmper* in E11.5 AGM explants after 24-h culture with BMP4 at displayed dose, without cytokines or serum normalized to *Tbp*. Experiments were performed twice. Error bars represent SD from the mean. Significance measured by Student’s *t* test: *, P = 0.035. (I) Expression of *Bmper* in OP9-BMP4 after reaggregation and culture with doxycycline to induce *Bmp4* overexpression. Expression was normalized to *Tbp*. For each condition, reaggregates were cultured in two separate wells in parallel. Error bars represent the SD from the mean (*n* = 2). Significance measured by Student’s *t* test: *, P = 0.026.

As Bmper has previously been described as a modulator of BMP signaling (Moser et al., 2003; Kamimura et al., 2004; Rentzsch et al., 2006; Moreno-Miralles et al., 2011; Helbing et al., 2013) we analyzed the spatiotemporal distribution of these factors in parallel. In agreement with our RNA-seq data, qRT-PCR shows that *Bmper* expression grows from negligible to a high level between E9.5 and E10.5 and further increases at E11.5, whereas *Bmp4* expression remains relatively high and steady over this period (Fig. 6 G). We then compared the localization of BMPER and BMP-activated cells (indicated by nuclear pSMAD1/5/8 immunostaining) within the AGM region (Fig. 6, A–C). Quantification of pSMAD1/5/8 signal within 80 µm of the aorta shows a significant reduction between E9.5 and E10.5 (Fig. 6, A–C and F; and Fig. S5 C). Consistently with the qRT-PCR analysis, BMPER increases within the AGM region at the protein level from E9.5 to E10.5 to E11.5 (Fig. 6, A–C). Notably, at E10.5 and E11.5, areas lateral to the dorsal aorta, with low BMPER signal, tend to have more pSMAD1/5/8-positive nuclei compared with ventral to the aorta, where the BMPER signal is higher (Fig. 6 D and Fig. S5 A). Both are practically undetectable in intra-aortic clusters (Fig. 6 E), although BMPER mRNA can be occasionally detected in some intra-aortic cells (Fig. 5 K). From these negatively correlated distributions, along with recent studies of the requirement for BMP4 inhibition for HSC maturation (Souilhol et al., 2016a), we propose that BMPER exerts its effect on HSC maturation through inhibition of BMP signaling. Interestingly, primordial germ cells, which are often in contact with regions expressing BMPER, also lack pSMAD1/5/8 (Fig. S5 B).

**Figure 7.**
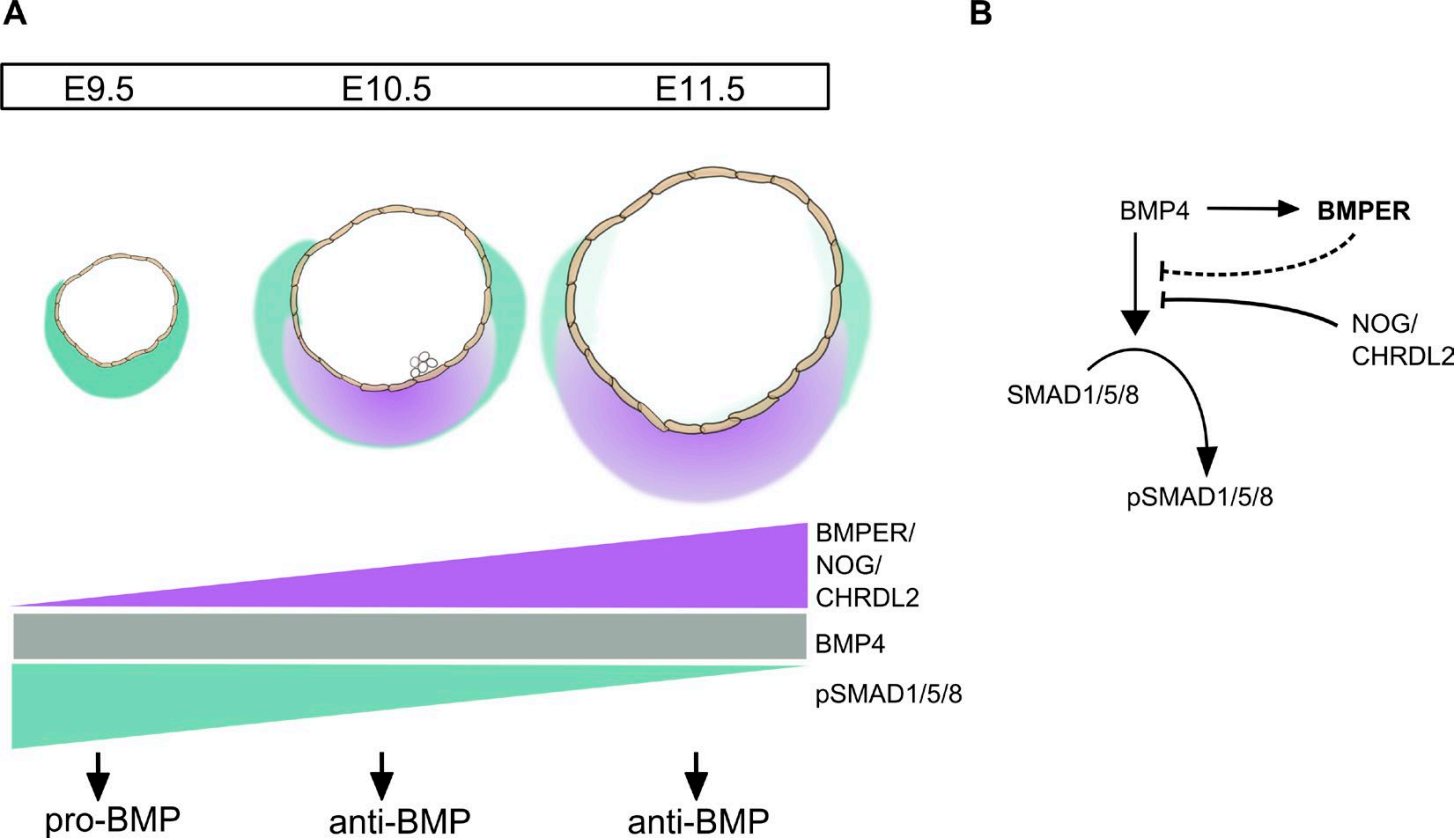
**The dynamic expression of BMP inhibitors and BMP4-dependent expression of *Bmper* mediate the switch to an anti-BMP environment in the AGM region.** (A) Schematic representation of the increasing ventral expression of BMP antagonists *Bmper*, *Noggin,* and *Chrdl2* in the AoV between E9.5 and E11.5, whereas the expression of *Bmp4* remains relatively constant. The balance of BMP agonists and antagonists therefore shifts to create an anti-BMP environment at E10.5 and E11.5 as seen by the reduction in pSMAD1/5/8 signal at these stages. (B) Schematic represents the action of BMP4 to drive phosphorylation of pSMAD1/5/8, which then translocates to the nucleus and promotes transcription. BMP4 also (through an unknown mechanism) promotes the transcription of *Bmper,* which, potentially in combination with Noggin, Chrdl2, and other inhibitors, inhibits the BMP4-driven phosphorylation of pSMAD1/5/8.

Analysis of the coexpression of *Bmp4* and *Bmper* across a range of cells types shows a positive correlation indicating a potential epistatic relationship (Fig. S5 D). Indeed, treatment of E11.5 AGM region explants with BMP4 and induction of *Bmp4* expression in OP9 cells both led to an increase in *Bmper* expression (Fig. 6, H and I), suggesting that BMP4 can drive *Bmper* expression. Thus, we propose that ventralized BMPER in the AGM region is induced by BMP4 signaling, and serves as a negative feedback response effector to restrict the BMP signaling in developing HSCs (Fig. 7).

## Discussion

The functional significance of the AGM region in HSC maturation has previously been described in detail, demonstrating the important acquisition of an autonomous supporting environment between E9.5 and E10.5 as well as the key dorso-ventral polarity (Taoudi and Medvinsky, 2007; Rybtsov et al., 2014, 2016; Souilhol et al., 2016a). Over this period, immature HSC precursors undergo active maturation and expansion, preferentially in the ventral domain of the AGM region (AoV). We hypothesized that RNA-seq of AGM subdomains through these critical developmental stages could yield insight into the factors that support this process. It has also been shown that the in vivo niche can be supplemented, to a degree, by OP9 cells after reaggregation (Rybtsov et al., 2011), suggesting that their transcriptional profile may refine the search for supportive factors. Here, producing a model of the full transcriptional landscape of these spatiotemporal transitions in the developing AGM region and OP9 cells in reaggregate culture enabled us to identify several secreted factors that may support HSC maturation. This is the first global gene expression analysis of these functionally demarcated regions of the developing HSC niche. The identification of previously unknown regulators of HSC development using functional screening by in vitro culture followed by transplantation demonstrates the utility of this dataset for gaining novel insight into the niche-dependent regulation of HSC development.

Our expression profiles agree with many previous observations about the AGM molecular landscape, such as the ventral polarization of Runx1, SCF, BMP4, and Noggin, as well as the dorsal polarization of Shh (North et al., 1999; Durand et al., 2007; Peeters et al., 2009; Ciau-Uitz et al., 2016; Souilhol et al., 2016a). Yet the breadth of this transcriptional approach has enabled us to capture a fuller picture of the signaling environment than has previously been possible. For example, at E10.5 the ventral polarization of BMP/TGF-β family ligands aside from *Bmp4*, encompassing *Bmp5*, *Bmp6*, *Inhbb*, *Inhba*, *Gdf3*, and *Tgfb2* as well as inhibitors *Smad6*, *Smad7*, *Noggin*, *Chrdl2*, and *Bmper*, indicates that several branches of the TGF-β superfamily may elicit effects in the HSC niche. In agreement with previous studies (Espín-Palazón et al., 2014; Li et al., 2014), we identified a proinflammatory signature encompassing IL-6, TNF and Jak/Stat in the E10.5 AoV environment and also several regulators of growth and differentiation (*Nell1*, *Igfbp3*, *Wnt2b*, and *Ibsp*). Overall, this analysis emphasizes the high molecular complexity of the developmental HSC niche.

Further transcriptional analysis showed that the reaggregated OP9 cells bear a closer resemblance to the E10.5 AoV niche than the conventional flat submersed culture, providing a potential explanation for the highly supportive properties of reaggregated OP9 cells. Among the common up-regulated genes in these two niches we find *Runx1*, a transcription factor essential for HSC development. *Runx1* is known to be expressed not only in hematopoietic lineages but also ventrally in the AGM subaortic mesenchyme adjacent to the endothelial floor (North et al., 1999), where its potential role in the non–cell-autonomous regulation of HSC emergence is yet to be elucidated.

The tractability of the reaggregate culture (Taoudi et al., 2008; Rybtsov et al., 2011, 2014, 2016) enabled higher throughput screening of candidate genes than many previous studies, where generation of transgenic or knockout mice poses a significant bottleneck. We functionally demonstrate that a relatively poorly characterized BMP pathway member, BMPER, significantly stimulates E9.5 proHSC maturation and, to a lesser extent, E11.5 preHSCs. BMPER, a known regulator of endothelial cell function (Moser et al., 2003; Heinke et al., 2008; Helbing et al., 2011), vascular patterning (Conley et al., 2000; Moser et al., 2007; Moreno-Miralles et al., 2011; Heinke et al., 2013; Dyer et al., 2014) and lung epithelium (Helbing et al., 2013), has never been shown to regulate HSC development. BMPER is reported to exhibit a biphasic control of BMP signaling, in low concentrations agonizing, and in high concentrations antagonizing BMP4 (Umulis et al., 2006; Serpe et al., 2008; Kelley et al., 2009). Here we show that BMPER protein is ventrally polarized in the AGM niche and its expression steeply grows during the E9.5–10.5 HSC maturation period, whereas active BMP signaling, manifested by nuclear staining of pSMAD1/5/8, declines. We observed spatial complementation rather than coincidence between BMPER- and pSMAD1/5/8-positive areas, indicating that BMPER acts as a BMP inhibitor in the E10.5 AGM region. Thus, although BMP4 and other BMP ligands are expressed in E10.5 AoV, a negative BMP signaling environment, possibly created through the concerted action of several inhibitory molecules including Noggin, facilitates HSC maturation (Souilhol et al., 2016a). Importantly, our in vitro experiments indicate that BMP4 can trigger *Bmper* expression, thus causing its own inhibition in the AGM region (Fig. 7). We found that perivascular cells immediately adjacent to the endothelial floor of the dorsal aorta are the strongest expressers of *Bmper* in the AGM region. Potentially, together with deeper low-expressing mesenchymal cells, they can serve an efficient barrier protecting intra-aortic clusters from BMP4, which can explain the lack of pSMAD1/5/8 nuclear accumulation in intra-aortic clusters.

Our model can explain a requirement for BMP4 at earlier stages of HSC development (Marshall et al., 2000; Wilkinson et al., 2009; Pouget et al., 2014), followed by a quick transition to BMP inhibition. A dynamic dependency on signaling pathways appears to be a common feature of HSC development, as has been shown previously with the loss of requirement for Notch (Gama-Norton et al., 2015; Lizama et al., 2015; Souilhol et al., 2016b) and Runx1 (Chen et al., 2009; Tober et al., 2013). Given the abundance of positive and negative BMP regulators in the AGM region, further investigation is required to explain how the entire network is spatially and functionally orchestrated during the multi-stepwise development of HSCs.

In conclusion, through a combination of genome-wide transcription analysis, bioinformatics approaches, and functional validation, we have identified novel regulators of HSC development. Although reaggregation culture significantly increases the throughput of candidate validation, the in vivo transplantation assay limits exhaustive screening approaches. This resource could yield other novel regulators of HSC development and facilitate the identification of the minimal conditions required to support HSC maturation in the absence of supportive cell stroma. To facilitate future studies, we have made these RNA-seq data available for the research community in an accessible graphical interface (http://agmniche.stembio.org/). A key future direction will be to deconvolve the complexity of the molecular landscape of the AGM regions and identify the degree to which different cell types contribute to this supportive signaling. Such insight will likely narrow the search for further functional screening of effectors and combinations thereof that regulate the HSC maturation process and ultimately inform protocols for the generation of HSCs from pluripotent cell sources.

## Materials and methods

### Animals

Embryos were obtained from mating C57BL/6 (CD45.2/2) mice. Day 0.5 was determined on the morning of discovery of a vaginal plug. Embryo stage was determined more accurately by number of somite pairs where E9.5 was attributed to embryos with 25–29 somite pairs (sp), E10.5 to embryos with 35–39 sp, and E11.5 to embryos with 41–45 sp. All experiments with animals were performed under a Project License granted by the Home Office (UK), University of Edinburgh Ethical Review Committee, and conducted in accordance with local guidelines.

### Embryo dissection and RNA extraction for RNA-seq

E10.5 embryos were subdissected into AoD, AoV, and UGR as described (Taoudi and Medvinsky, 2007; Souilhol et al., 2016a) and E9.5 subdissected into AoD and AoV in a similar way. AGM subdomains were pooled from between 15 and 34 embryos in three separate experiments. The total RNA was extracted from these pools with an RNeasy minikit (QIAGEN) including DNase I treatment (QIAGEN). RNA quality measured with Agilent 2100 Bioanalyzer (Agilent) to ensure all samples had RNA integrity number >9.

### RNA-seq and analysis

RNA-seq libraries were prepared with a TruSeq RNA Library Preparation kit (Illumina) and multiplexed. From these libraries, 50 base single-end sequence reads were generated with Illumina HiSeq 2500 (Illumina), yielding 50 million reads per sample. For sequencing OP9, the same platform was used, but sequencing yielded 10 million reads per sample. Library preparation and cDNA sequencing was performed in the Edinburgh Genomics facility at the University of Edinburgh.

Sequencing reads were processed using the RNA-seq analysis pipelines of GeneProf (Halbritter et al., 2011). In brief, sequencing reads were aligned to mouse genome NCBI37/mm9 with TopHat (Trapnell et al., 2009) v1.2.0 (2012-11-14) and Bowtie (Langmead et al., 2009) v0.12.3 (2012-04-10). The mRNA levels per gene were quantified by a custom script in Geneprof, which accounts for multimapping reads. In brief, all uniquely mapped reads overlapping with each annotated gene (Ensembl 58 Mouse Genes, NCBIM37) were summed, whereas ambiguously mapped genes were divided into fractions, weighted proportionally to the number of uniquely mapped genes in each possible gene assignment. Read counts were then scaled per million (RPM). Downstream analysis of normalized read counts used the R statistical programming language and environment (R version 3.2.2, 2015–08-14, R Core Team, 2015). To reduce gene expression noise, genes with expression intensity < 0.5 RPM in all samples were removed. Samples were plotted based on the principal components calculated from the remaining “expressed" genes with R stats “prcomp,” where variables were zero-centered and scaled to unit variance. The association of categorical traits with principal components was tested by one-way ANOVA, considering AoV and AoD as factors of “dorso-ventral polarity” and E9.5 and E10.5 as factors of “embryonic stage.”

Gene clusters were calculated using the R package ConsensusClusterPlus (Wilkerson and Hayes, 2010), version 1.22.0. The top 3000 dynamically regulated genes (measured by coefficient of variation), expressed >0.5 RPM in at least one sample, were median-centered, then used as input. K clusters were calculated using the hierarchical clustering of Pearson distance. Clustering was iterated 50 times for K clusters in the range 2 to 10, and K = 5 was selected as the lowest number of clusters for which the cumulative distribution function reaches a maximum. The significant association of mean expression level of genes belonging to each cluster with categorical values “E10.5 AoV,” “E10.5 AoD,” “E10.5 UGR,” “E9.5 AoV,” and “E9.5 AoD” was calculated with ANOVA. Gene ontology enrichment was calculated for each gene cluster using the R package TopGO (Alexa and Rahnenfuhrer, 2016), using all expressed genes in the samples as a background and filtered by weighted Fisher’s exact test.

Differentially expressed genes were calculated by applying negative binomial distribution with the R/Bioconductor package DESeq (Anders and Huber, 2010; 2011-03-15) within GeneProf and selecting those with fold change increase >2 and p-value <0.05 after adjustment for multiple hypothesis correction by the Benjamini and Hochberg procedure (Benjamini and Hochberg, 1995). Comparisons were between groups of replicates from each AGM tissue. For OP9 samples, comparisons were between cells that had been cultured in flat submersed conditions and cells that had been reaggregated, as IL-3 treatment had no effect on transcription and variance from the OP9 cell source was equivalent to variance due to passage number. Secreted factors were defined as those annotated with either gene ontology “GO:0005576” (extracellular region), “GO:0005615” (extracellular space).

Relative pathway enrichment was calculated using the Limma package in R/Bioconductor (Ritchie et al., 2015). In brief, the trimmed mean of M values scale normalization method (Robinson and Oshlack, 2010) was applied to read counts followed by mean-variance modeling at the observational level (voom) using an experiment design matrix (Law et al., 2014). Pathway enrichment was calculated with rotation gene set testing (ROAST), which uses a Monte Carlo simulation technology instead of permutation (Wu et al., 2010). Comparative enrichment analysis was with canonical signaling pathways that have been previously implicated in the regulation of hematopoiesis, comprising proinflammatory signals including IFN, TNF, and NF-κB (Espín-Palazón et al., 2014; Li et al., 2014; Sawamiphak et al., 2014; González-Murillo et al., 2015); ILs (Robin et al., 2006; Taoudi et al., 2008), VEGF (Kabrun et al., 1997; Gering and Patient, 2005; Burns et al., 2009; Lugus et al., 2009; Ciau-Uitz et al., 2013; Leung et al., 2013), PDGF (Levéen et al., 1994; Soriano, 1994; Chhabra et al., 2012), Hedgehog (Peeters et al., 2009; Wilkinson et al., 2009; Souilhol et al., 2016a), Notch (Gering and Patient, 2010; Kim et al., 2014; Gama-Norton et al., 2015; Souilhol et al., 2016b), SCF/Kit (Ding et al., 2012; Rybtsov et al., 2014), Wnt (Trompouki et al., 2011; Ruiz-Herguido et al., 2012; Sturgeon et al., 2014), TGF-β (Nimmo et al., 2013; Blank and Karlsson, 2015; Monteiro et al., 2016), BMP (Durand et al., 2007; Crisan et al., 2015; Souilhol et al., 2016a), and Nodal (Nostro et al., 2008; Sturgeon et al., 2014; Pina et al., 2015) which were obtained from the Molecular Signatures Database (Subramanian et al., 2005; Fig. 2 A and Table S3). To ensure the search was comprehensive, we included all available annotations of these pathways from different databases such as KEGG (Kanehisa et al., 2016), BioCarta (Nishimura, 2001), PID (Schaefer et al., 2009), and Reactome (Croft et al., 2014; Fabregat et al., 2016). Pathways were mapped from human to mouse with a homology file from MGI HOM_MouseHumanSequence.rpt. Significant pathways were determined as those with multiple hypothesis corrected p-values <0.2. Genes contributing to pathway enrichment were determined from differentially expressed genes (using the same linear model applied with Limma ROAST) with uncorrected p-values <0.2, because of the contribution of multiple genes to the pathway enrichment scores. Significant overlaps between OP9 differentially expressed genes and AGM clusters were calculated by hypergeometric distribution (R phyper) where the number of genes in the intersection represents *k* successes in *n* draws (where *n* is the number of genes in a cluster), from a population *N* (where *N* represents the total number of genes expressed in OP9 cells) that contains *K* successes (where *K* represents the number of significantly up-regulated genes in reaggregated OP9 cells).

Meta-analysis was with datasets obtained from the sequence read archive (Leinonen et al., 2011): SRP033554 (Huang et al., 2014), SRP049826 (Solaimani Kartalaei et al., 2015), SRP045264 (Lara-Astiaso et al., 2014), SRP036025 (Kemp et al., 2014), SRP023312 (Pereira et al., 2013), SRP026702 (Kunisaki et al., 2013), ERP001549 (Magnúsdóttir et al., 2013), and AGM and OP9 datasets generated here through the automated import tool in GeneProf (Halbritter et al., 2011), and processed with GeneProf as above. Read counts for each experiment were merged in R and normalized with DEseq2 (Love et al., 2014) version 1.8.2 using treatment condition as a factor for variance stabilizing transformation. Correlation between genes was with Spearman’s rank correlation.

### OP9 cell culture and reaggregation

OP9 cells were maintained in IMDM (Invitrogen), 20% FCS supplemented with l-glutamine (4 mM), penicillin/streptomycin (50 U/ml) and passaged every 4–5 d. For RNA-seq, cells grown in flat culture had previously been maintained in the A. Medvinsky or the M. de Bruijn group. For reaggregate culture, a single cell suspension was generated by adding trypsin and then centrifuged at 430 *g* for 12 min in 200-µl pipette tips sealed with parafilm to form a pellet. Reaggregated cells were cultured at the liquid–gas interface on 0.8-µm nitrocellulose filters (Millipore) at 37°C in 5% CO_2_ on IMDM (Invitrogen), 20% FCS supplemented with l-glutamine (4 mM), penicillin/streptomycin (50 U/ml), and with or without IL-3 (200 µg/µl) for 48 h. Reaggregates were harvested and cells dissociated with collagenase/dispase, then RNA extracted.

For generating *Bmp4* doxycyline-inducible OP9 cells, *Bmp4* cDNA was cloned into a doxycycline inducible bicistronic expression vector pPBhCMV1-cHA-IRESVenuspA (gift from H. Niwa, Institute of Molecular Embryology and Genetics, Department of Pluripotent Stem Cells, University of Kumamoto, Kumamoto, Japan). In this construct, both *Bmp4* and *Venus* were expressed upon induction with doxycycline. 100,000 OP9 cells were transfected with this construct by electroporation using a NEON transfecting system (Invitrogen). 24 h after electroporation, cells were cultured with 1 µg/ml doxycycline (Clontech), and the Venus-positive population was sorted. After a week, in the absence of doxycycline induction, the Venus-negative population was sorted and maintained. For induction of *Bmp4* followed by RNA extraction, cells were cultured in OP9 media with 1 µg/ml doxycycline for 24 or 48 h.

### HSC maturation ex vivo

For ex vivo reaggregate cultures, caudal parts were dissected from E9.5 embryos, and from E11.5 embryos the AGM region was dissected, then subdissected into AoV and AoD. Dissected embryonic tissues were dissociated by collagenase/dispase and then either self-reaggregated (E11.5 tissues) or coaggregated with OP9 stromal cells (E9.5 tissues). For self-reaggregation, AGM cell suspensions were centrifuged at 430 *g* for 12 min in 200-µl pipette tips sealed with parafilm to form a pellet. For coaggregation with OP9 cells, suspensions of 1 e.e. of embryo cells were mixed with 10^5^ OP9 cells before centrifugation. Cell aggregates or explants were cultured at the liquid–gas interface on 0.8-µm nitrocellulose filters (Millipore) at 37°C in 5% CO_2_ for either 5 d (with E11.5 cells) or 7 d (with E9.5 cells). For E9.5 reaggregates, the culture media was IMDM (Invitrogen), 20% FCS supplemented with l-glutamine (4 mM), 50 U/ml penicillin/streptomycin, 100 ng/ml SCF, 100 ng/ml IL-3, and 100 ng/ml FLT3L (all purchased from Peprotech). 2 ml of culture media was added for the first 24 h, and then this was replaced with 5 ml fresh media for the rest of the culture period. For the E11.5 aggregates, the culture media was 5 ml IMDM alone. Additional recombinant proteins were GDF-3, CHRDL2, BMPER, INHBB, IBSP, IGFBP3, NELL1 and WNT2b (R&D Systems); CXCL10 and CCl4 (Peprotech). After the stated culture period, the whole membrane was immersed in collagenase/dispase (Roche) for 40 min at 37°C to remove reaggregates and dissociate them into a single cell suspension.

### Long-term repopulation assay

Donor cells were injected intravenously into C57BL/6 CD45.1/2 sublethally irradiated (1,150 rad) mice along with 20,000 C57BL/6 CD45.1/1 bone marrow carrier cells. The injected dose of cells is stated in the text per embryo equivalent, i.e., unit of cells equivalent to the number present in one embryo. The transplanted dose was adjusted depending on the culture system and regularly calibrated based on similar reaggregate experiments performed in the laboratory around the same time, so that small numbers of HSCs would be detected, but to ensure controls did not reach a saturating level of repopulation.

To detect long-term hematopoietic repopulation, peripheral blood was collected 16 wk after transplantation by bleeding the tail vein into 500 µl of 5 mM EDTA/PBS. Erythrocytes were depleted using PharM Lyse (BD), and cells were stained with anti-CD16/32 (Fc-block), anti–CD45.1-APC (cloneA20, eBioscience), and anti–CD45.2-PE (clone 104, eBioscience) mAbs. The percentage of donor CD45.2 cells was analyzed using FACSCalibur and flowJo software (TreeStar).

Multilineage contribution was detected from peripheral blood between 12–16 wk after transplantation. In brief, contribution to all blood lineages was detected by exclusion of recipient CD45.1^+^ cells and staining with lineage-specific monoclonal antibodies for Mac1, CD3e, CD4, CD41, Gr1, B220, CD8, and Ter119. Antibodies were conjugated with PE, FITC, APC, or biotin, and cells were analyzed using FACSCalibur and flowJo software (TreeStar). Significant differences in contribution were calculated with the Wilcoxon rank-sum test (R “stats” package), and correction for multiple testing was performed with the Benjamini-Hochberg procedure (Benjamini and Hochberg, 1995) using a false discovery rate (FDR) threshold of 10%.

### CFU-C assay

After reaggregate culture of E9.5 caudal part, OP9, and cytokines, cells were dissociated and plated in methylcellulose (MethoCult3434 medium; STEMCELL Technologies) at a concentration of 0.005 e.e. of the starting culture. This concentration of cells was used to avoid saturation of colonies. After 7 d, granulocyte-macrophage progenitor; granulocyte, erythrocyte, monocyte, megakaryocyte; and erythroid burst-forming unit colonies were counted and normalized per embryo equivalent.

### Immunostaining and microscopy

Embryos were fixed in 4% paraformaldehyde at 4°C overnight then embedded in gelatin by first incubating them in 15% sucrose for 2 h at 4°C, followed by PBS/15% sucrose/7% gelatin at 37°C, followed by flash freezing in liquid nitrogen. Transverse sections of 7 µm were cut with CM1900 Cryostat (Leica). Sections were permeabilized with PBS/0.5% Triton X-100 for 10 min, then blocked for 30 min with PBS/10% FCS. Antibodies were diluted in PBS/2% FCS. For pSMAD1/5/8 staining, blocking was with PBS/10% serum/1% BSA/0.1% Triton X-100, and antibodies were diluted in PBS/5% serum/1% BSA/0.1% Triton X-100. Primary staining was with rat anti-CD31 (1:100, MEC13.3; PharMingen), goat anti-BMPER (1:100, AF2299; R&D Systems), rabbit anti-RUNX1 (1:200, clone EPR3099; Abcam), anti–SSEA-1–biotin (1:1000, MC-480; eBioscience), sheep anti-CD146 (1:100, AF6106; R&D Systems), or rabbit anti–P-Smad1/5/8 (1:100, clone D5B10; Cell Signaling Technology) overnight followed by incubation with anti–goat NL577 (1:100; R&D Systems), anti–rabbit Alexa Fluor 488 (1:100; Invitrogen), anti–rat Alexa Fluor 488 (1:500; Invitrogen) anti–rabbit Alexa Fluor 647 (1:500, Abcam), or streptavidin-PE (1:500, PharMingen) for 2 h, followed by counterstaining with DAPI. Images were acquired with an inverted confocal microscope (SP8, Leica) 63× objective at room temperature and processed using ImageJ (National Institutes of Health). For BMPER control images (Fig. S3) and pSMAD1/5/8 quantification (Fig. S5 C), acquisition was with upright widefield (BX61; Olympus) with 20× objective at room temperature.

### In situ hybridization

For probe preparation for DIG in situ hybridization, Bmper-CV2 plasmid (Coffinier et al., 2002) was linearized with EcoRI restriction enzyme and transcribed by T7 RNA polymerase to synthetize DIG-labeled single-stranded antisense RNA probe.

For DIG-labeled in situ hybridization, paraffin sections were dewaxed and dehydrated, and washed in PBS. Sections were permeabilized with proteinase K treatment (7 µg/ml) for 25 min, fixed in 4% PFA, washed in PBS, then incubated in acetic anhydride in triethanolamine. Slides were washed in PBS, dehydrated, and air-dried. Probe was denatured, then applied at 1 µg of probe/ml hybridization buffer to slides, covered with coverslips, and hybridized at 65°C overnight. Slides were washed in 50% formamide and 5xSSC, pH 4.5 at 65°C. Unhybridized probe was digested with RNase A (20 µg/ml) and washed in 2xSSC and TBST. Sections were blocked with 1x Animal-Free Blocker (Vector Labs) for 1 h at room temperature, and potential endogenous alkaline phosphatase activity was blocked by Bloxall (Vector Labs) for 10 min at room temperature. Anti–DIG-Fab fragment antibody (1:2,000; Roche) in blocking solution was applied on slides and incubated overnight. Antibody was washed off with TBST, slides were incubated in NTMT pH 9.5 solution, and color was developed in BM Purple (Roche) in the dark, 4°C overnight. Slides were washed in PBS and ddH2O, dehydrated, air-dried, and mounted with VectaMount (Vector Labs).

### Fluorescence-activated cell sorting

To sort stromal cells or preHSCs, AGM regions were dissected from E10.5 embryos and dissociated with dispase/collagenase. Then single-cell suspensions were stained with Ter119-FITC (1:100, TER-119, eBioscience), anti–CD45-V450 (1:100, 30-F11; BD Horizon), anti–VE-cadherin–e660 (1:100, eBio BV13; eBioscience), and anti–CD146-PE (1:400, ME-9F1; BioLegend); or lineage (Ter119, Gr1, CD3e, CD11b all PerCP_Cy5.5 conjugated from eBioscience), CD45-V500 (1:100, 30-F11; BD Pharmigen), VE-cadherin–e660 (1:100, eBioBV13; eBioscience), CD43-bio (1:100, eBioR2/60; eBioscience), CD41-BV421 (1:200, MWReg30; BioLegend), CD146-PE (1:400, ME-9F1; BioLegend), and secondary antibody streptavidin-BV650 (1:100; BioLegend). Finally, 7-Aminoactinomycin D viability staining solution was added for exclusion of dead cells. FACSAriaII and FACSDiva software (BD Bioscience) were used for sorting, and gates were set using appropriate fluorescence-minus-one controls.

### cDNA preparation and qRT-PCR

For qRT-PCR from bulk cultures, RNA was extracted with Qiagen RNeasy microkit or minikit (QIAGEN), and cDNA was prepared with SuperScript III reverse transcription and random hexamer primers (Invitrogen). qRT-PCR was performed using LightCycler 480 SYBR Green I MasterMix (Roche) for detection. For detection in small populations of cells, up to 200 cells were directly sorted by FACS into 10 µl of 2 × Reaction Mix (CellsDirect; Invitrogen) and 0.2 µl RNase inhibitor (SUPERase-In Ambion AM2694). Superscript III/Taq mix (CellsDirect) and gene-specific primers (10 µM each) were added to the cell lysate to directly reverse-transcribe and amplify cDNA (PCR program: 50°C for 15 min; 95°C for 2 min; 18 cycles of 95°C for 15 s; 60°C for 4 min). Control samples that underwent the Taq amplification in the absence of SuperScript III reverse transcription were used to assess contamination or amplification of genomic DNA. In this case, the Lightcycler 480 probes mastermix kit (Roche) was used for qRT–PCR detection on diluted cDNA. All qRT-PCR used two or more biological replicates, and expression was measured relative to TATA-binding protein (*Tbp*) and, where indicated, all scaled to one sample. Significant differences were measured by the two-tailed Student’s *t* test on values that had been normalized only to *Tbp.* Correction for multiple testing was with Benjamini-Hochberg procedure (Benjamini and Hochberg, 1995) and significant differences displayed only if the false discovery rate was less than 10%. Primer sequences can be found in Table S7.

### Accession number

The data discussed in this publication have been deposited in NCBI’s Gene Expression Omnibus (Edgar et al., 2002) and are accessible through GEO Series accession no. GSE102859.

### Online supplemental material

Fig. S1 provides full details of the analysis to determine stable gene clusters from the AGM RNA-seq data. Table S1 gives the association scores of the principal components from the RNA-seq data and their associated categorical traits. Table S2 lists the gene members of the clusters defined in (Fig. 1). Table S3 gives the full list of hematopoiesis-related signaling pathways that were tested for enrichment in E10 AoV. Table S4 gives the pathways enriched in OP9 cells cultured in reaggregate conditions, and Table S5 gives the genes common to E10 AoV (cluster4) and up-regulated in OP9 after reaggregation. Fig. S2 gives the repopulation and colony-forming assays from the functional screen of effectors of HSC maturation, and Table S6 gives the corresponding p-values for every comparison of repopulation results for all recombinant protein treatments versus controls. Fig. S3 gives the positive and negative controls for BMPER immunostaining and in situ hybridization. Fig. S4 shows the sorting strategy for defining subpopulations of the AGM region and the expression of surface markers and *Bmper* in OP9 cells. Fig. S5 gives higher magnification views of SMAD1/5/8 and BMPER costaining in the AGM region, shows how pSMAD1/5/8 staining was quantified, and shows the correlation of *Bmp4* and *Bmper* across RNA-seq data from several cell types. Table S7 gives the primers used throughout the study.

## Supplementary Material

Supplemental Materials (PDF)

Tables S1-S7 (zipped Excel files)
