# Supplementary material for: A molecular roadmap of the AGM region reveals BMPER as a novel regulator of HSC maturation
Source: J Exp Med. 2017 Dec 4;214(12):3731–51. doi: 10.1084/jem.20162012 (PMC5716029; doi:10.1084/jem.20162012)
Supplement: Supplemental Materials (PDF) [file JEM_20162012_sm.pdf]

The Journal of Experimental Medicine

SUPPLEMENTAL MATERIAL

McGarvey et al., https://doi.org/10.1084/jem.20162012

A

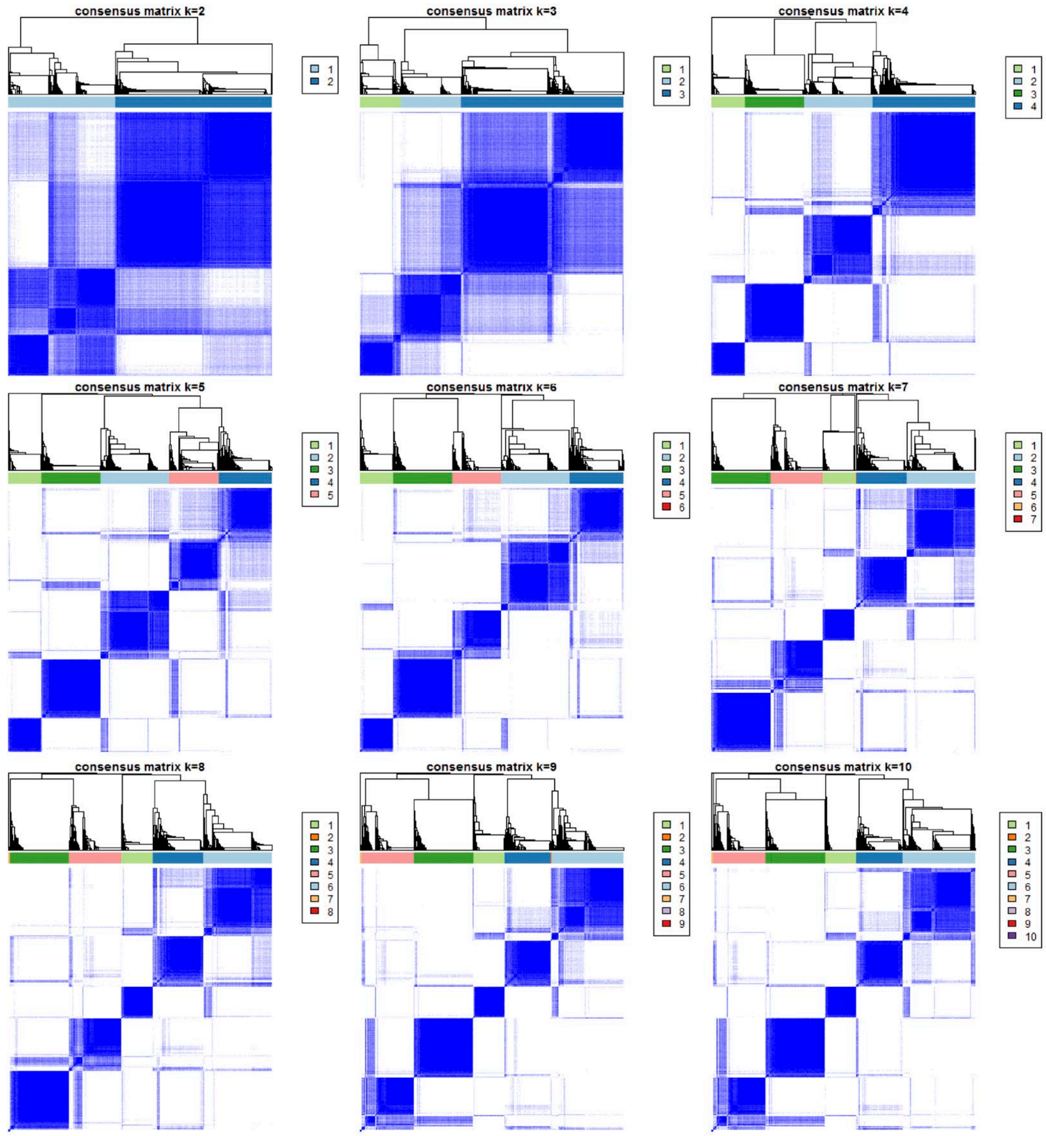

B C

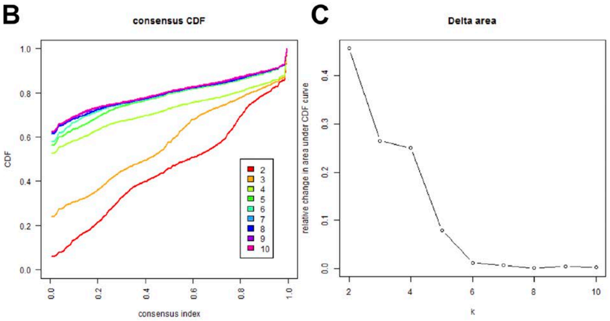

D

Cluster mean-trait ANOVA

Phenotype	Cluster1	Cluster2	Cluster3	Cluster4	Cluster5
E9AoD	2.074e-08	0.1846391938	0.39591550817	0.03239630564	0.18156455637
E9AoV	0.56151425276	0.07573289664	1e-11	0.87301681336	0.38609987268
E10AoD	0.66339921718	1.38501e-06	0.21270901452	0.35187078796	0.12252566908
E10AoV	0.14499340665	0.70698537093	0.65066019743	4.7232e-07	0.75537957842
E10UGR	0.31746372672	0.49749069351	0.31108359545	0.79367840296	3.3854e-07

Figure S1. **Defining stable clusters for partitioning genes into molecular signatures.** (A) Consensus clustering of the 3,000 most dynamically expressed genes setting K clusters in the range 2–10 shows most stable partitioning of genes. K in the range 2–10, clusters partitions are depicted by a consensus matrix heat maps where rows and columns represent unique genes and the color scales represents consensus values, which are the proportion of times that two items occupied the same cluster out of 50 iterations. Color keys indicate cluster definitions. (B) Cumulative distribution function of clustering in setting K in the range 2–10. (C) Delta area under curve of clustering for K in the range 2–10 to determine the relative increase in cluster consensus with every increase in K. (D) Table shows association of cluster means with categorical sample phenotypes by ANOVA.

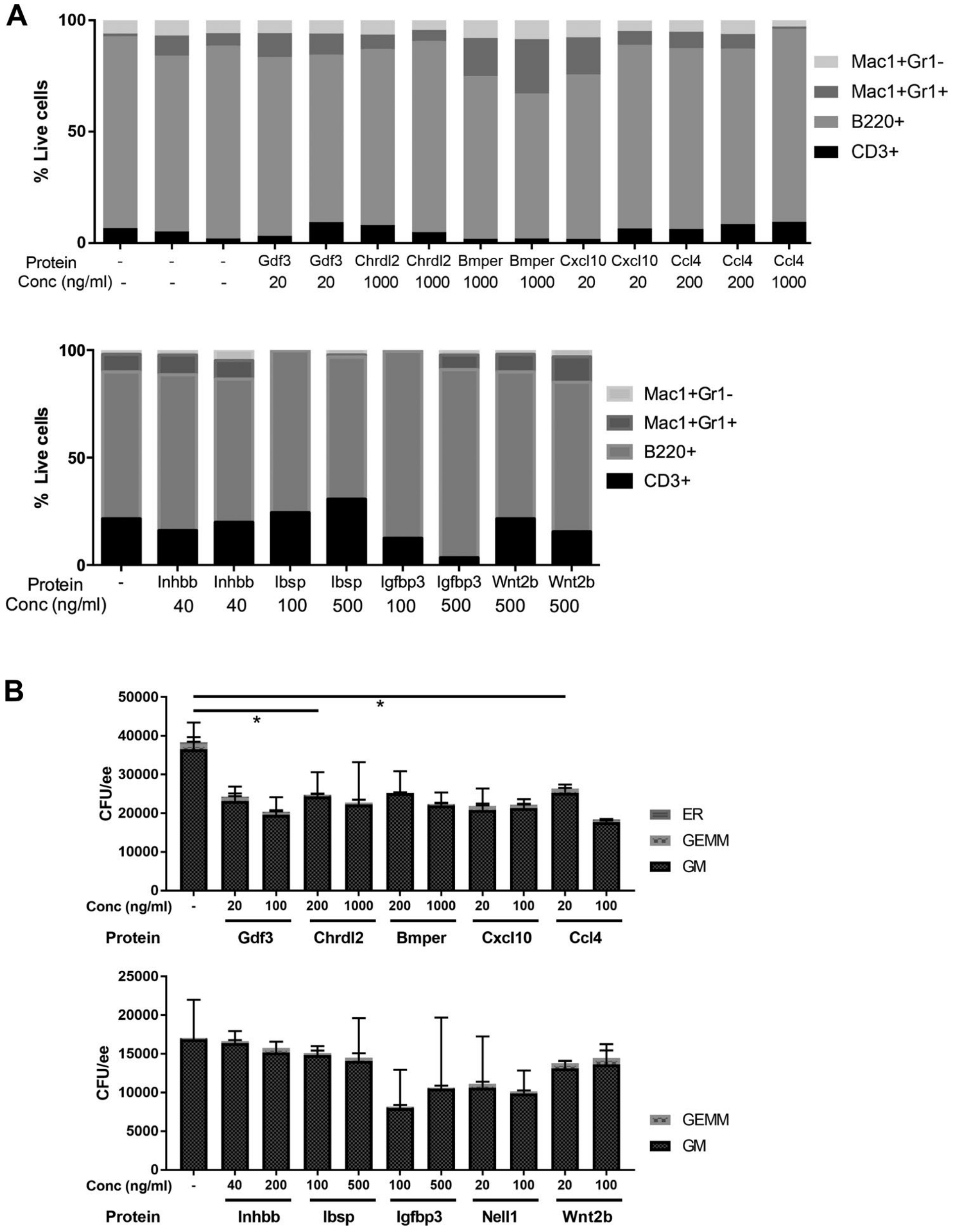

Figure S2. **Multilineage repopulation of mice transplanted with HSCs matured in culture with recombinant proteins and colony-forming assays.** (A) Quantification of multilineage contribution from repopulated mice representing each treatment condition. Populations are a percentage of live donor cells ($CD45.2^+$) from peripheral blood of transplanted mice: myeloid ($Mac1^+Gr1^+$ and $Mac1^+Gr^-$), B cells ($B220^+$), and T cells ($CD3^+$). (B) Colony-forming assays from E9.5 caudal parts reaggregated and cultured with OP9 cells, SCF, IL-3, FLT3L, and recombinant proteins for 7 d, followed by 7-d culture in methylcellulose. ER, proerythroblast type colonies; GEMM, granulocyte/erythrocyte/monocyte/megakaryocyte; GM, granulocyte/monocyte. Each methylcellulose culture was with 0.005 e.e., with two technical replicates and two biological replicates. Error bars represent SD from the mean. Significance calculated by *t* test: *, Ccl4 100 ng/ml treatments, P = 0.034; Chrdl2 200 ng/ml treatment, P = 0.039.

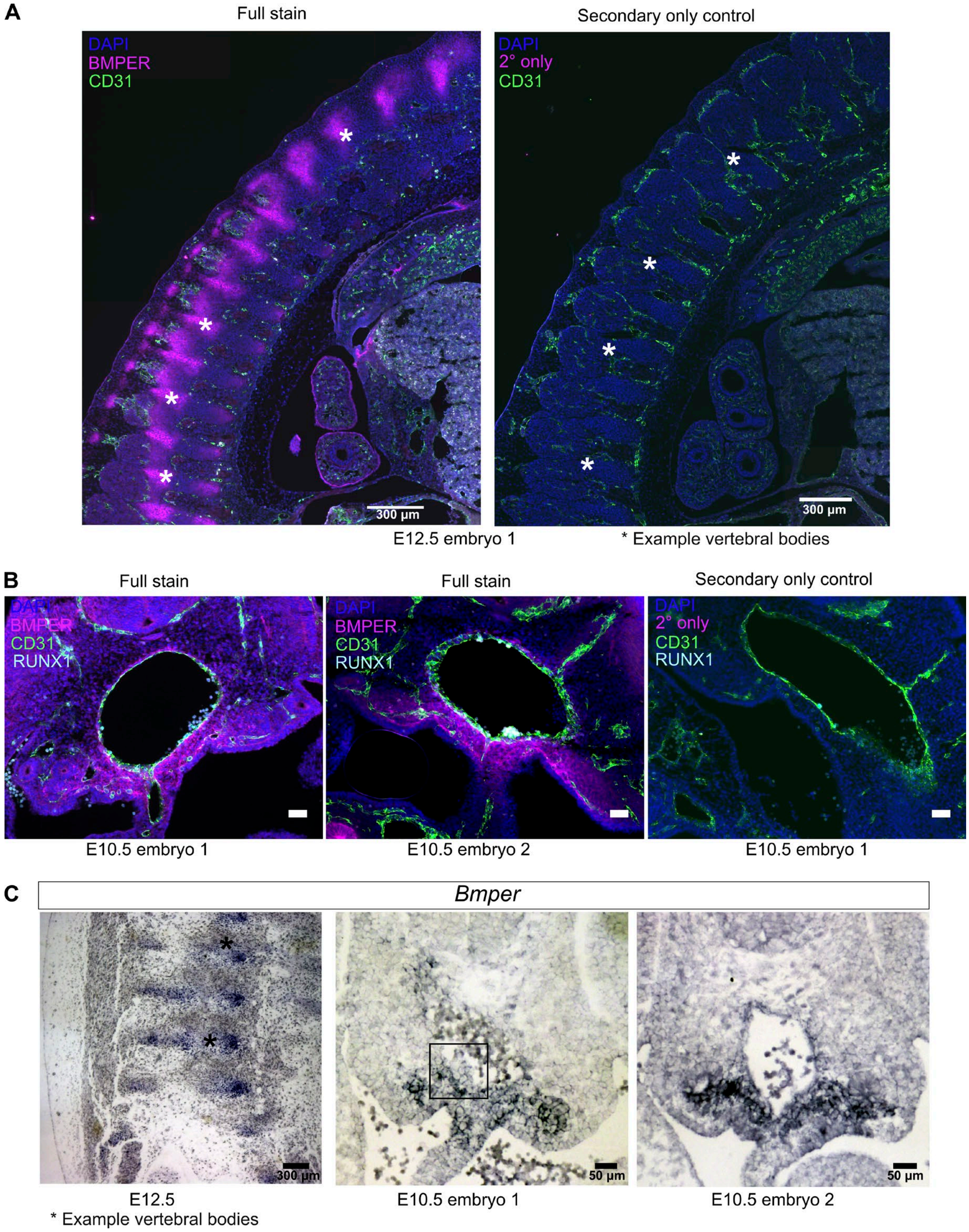

Figure S3. **Defining protein level and mRNA level staining for BMPER.** (A) Distribution of BMPER protein in vertebral bodies in E12.5 sagital sections measured by anti-BMPER immunostaining in agreement with Zakin et al. (2008). Green, CD31; magenta, BMPER; blue, DAPI. Bars, 300 µm. (B) Distribution of BMPER protein in E10.5 AGM transverse sections from two different embryos measured by immunostaining and control lacking the primary BMPER antibody. Green, CD31; magenta, BMPER; cyan, RUNX1; blue, DAPI. Bars, 50 µm. (C) Staining for *Bmper* mRNA in control E12.5 section of vertebral bodies, and two transverse sections of the E10.5 AGM region from two different embryos with box highlighting intra-aortic cluster shown in Fig. 5 L. Bars: (left) 300 µm; (middle and right) 50 µm.

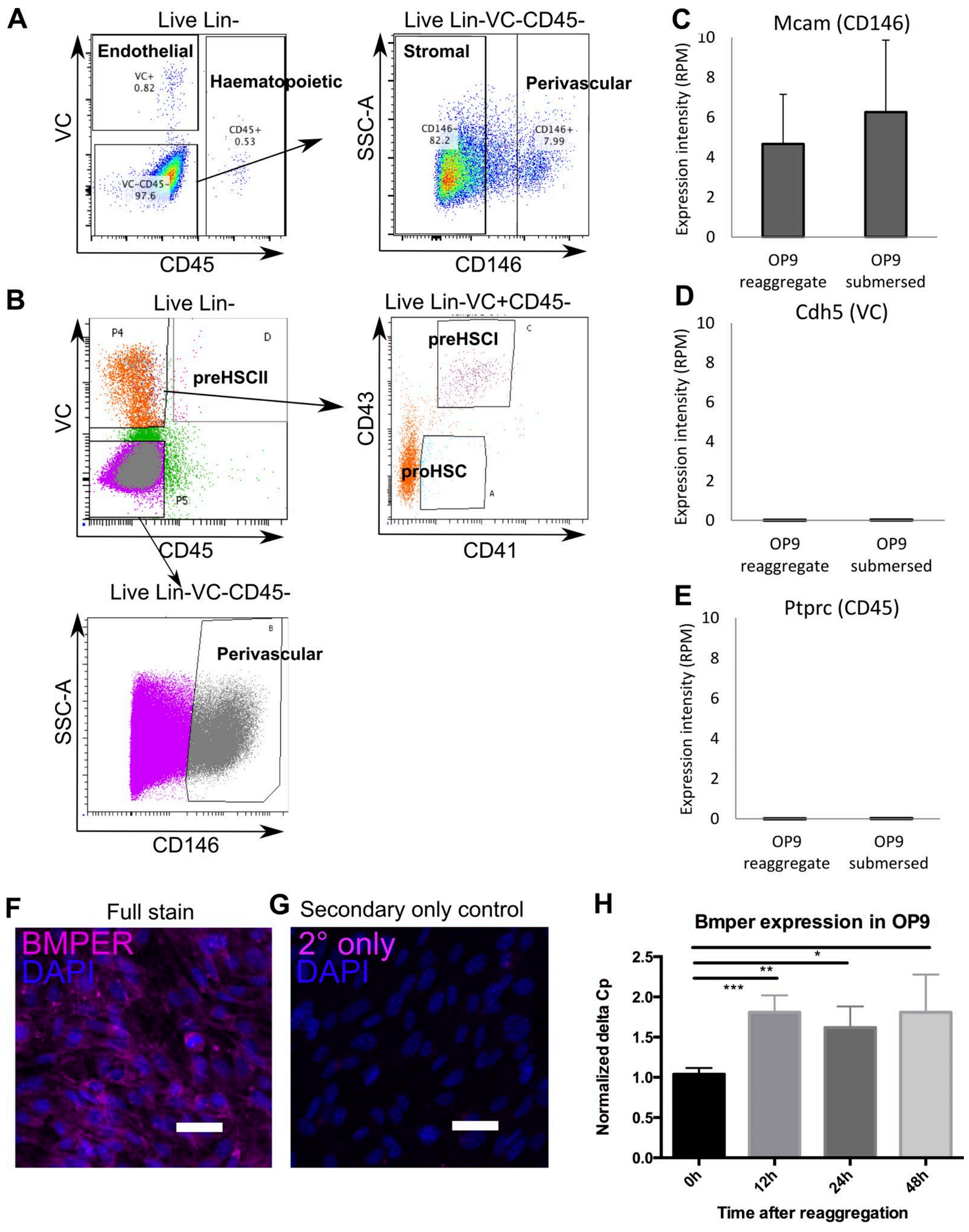

Figure S4. **Sorting subpopulations of the AGM region and defining BMPER in OP9 cells.** (A) Sorting strategy for isolation of AGM stromal populations: $Lin^-VC^-CD45^+$, representing hematopoietic cells; $Lin^-VC^+CD45^-$, endothelial cells; $Lin^-VC^-CD45^-CD146^+$, putative perivascular cells; and $Lin^-VC^-CD45^-CD146^-$, remaining stroma. Representative plots for three experiments. (B) Sorting strategy for isolation of AGM HSC precursor lineage populations: $Lin^-VC^+CD45^-CD43^-CD41^{lo}$ proHSC, $Lin^-VC^+CD43^+CD41^+$ type I preHSC, $Lin^-VC^+CD45^+$ type II preHSC, and $Lin^-VC^-CD45^-CD146^+$ putative perivascular cells. Representative plots for two experiments. Gates were defined by fluorescence-minus-one (FMO) control samples. (C–E) Expression of *Mcam* (CD146; C), *Cdh5* (VC; D), and *Ptprc* (CD45; E) in OP9 cells cultured in submersed conditions or in reaggregate culture for 48 h. Expression intensity is in RPM from RNA-seq data. (F) Intracellular and secreted BMPER from OP9 cells grown in submersed culture and costained with anti-BMPER. Magenta, BMPER; blue, DAPI. Bar, 50 µm. (G) Secondary antibody-only control for intracellular and secreted BMPER from OP9 cells grown in submersed culture. Magenta, secondary antibody; blue, DAPI. Bar, 50 µm. (H) *Bmper* transcript levels measured in OP9 after reaggregation for 0, 12, 24, and 48 h. Measured by qRT-PCR relative to *Tbp*. Error bars represent the SD from the mean of two independent experiments with two technical replicates each. ***, $P = 0.0005$; **, $P = 0.006$; *, $P = 0.02$.

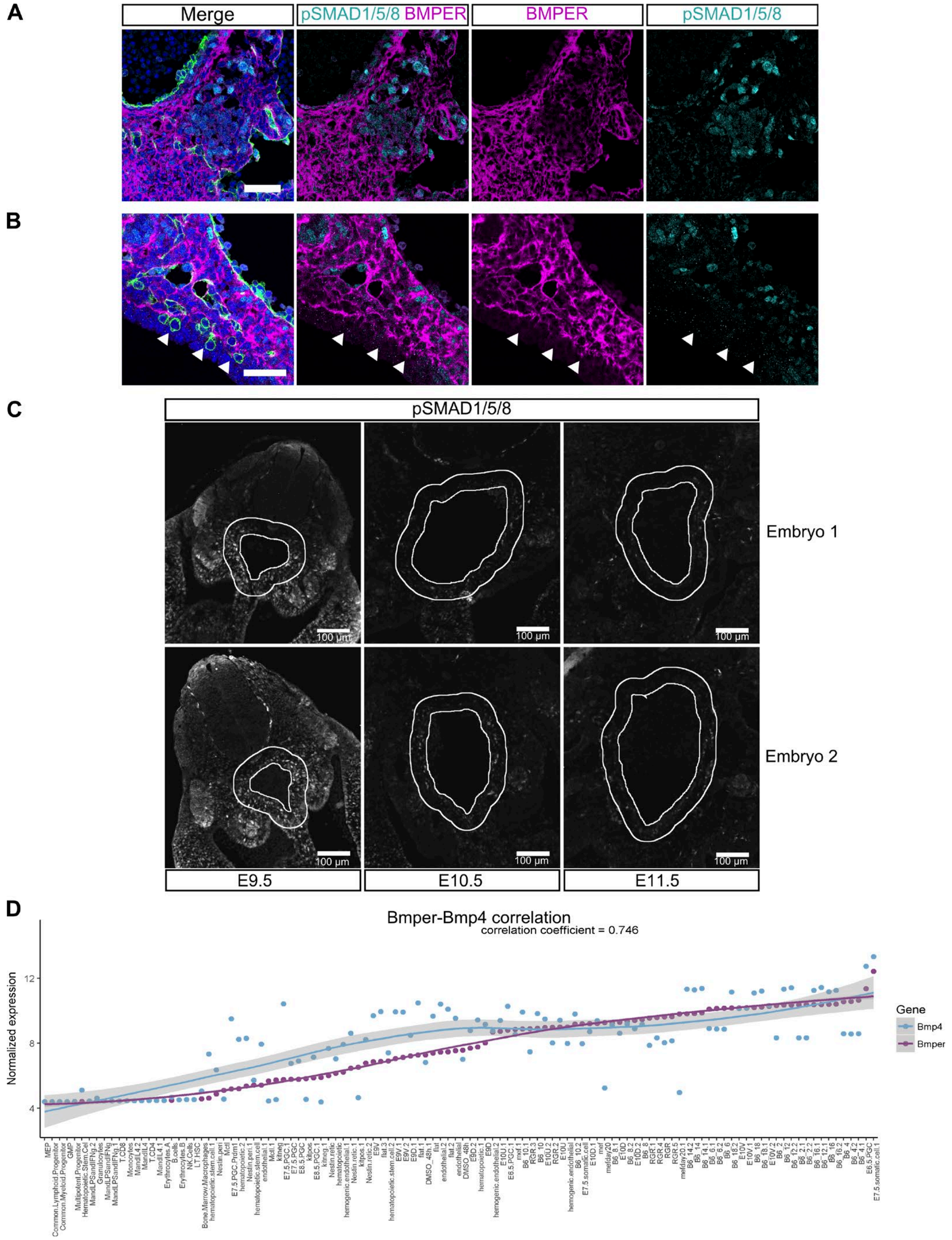

Figure S5. **BMPER-pSMAD1/5/8 complementarity and quantifying the protein level of pSMAD1/5/8 around the dorsal aorta from E9.5 to E11.5.** (A) Higher magnification view of E11.5 ventro-lateral region highlighted in box of Fig. 6 C. Magenta, BMPER; cyan, pSMAD1/5/8; green, CD31; blue, DAPI. Dotted line indicates the boundary around regions with high BMPER protein. (B) Higher magnification view of E10.5 ventro-lateral region from Fig. 6 B. Magenta, BMPER; cyan, pSMAD1/5/8; green, CD31; blue, DAPI. Arrowheads indicate CD31-positive primordial germ cells. (C) Example transverse sections of the AGM from embryos stage E9.5, E10.5, and E11.5 used for quantification of pSMAD1/5/8 presented in Fig. 6 F. For every section, the inner band was defined by the CD31 staining on the luminal side of the endothelium, and the outer band was added 80 µm around the first band. The mean gray value was calculated within these regions for every section. (D) Normalized expression of *Bmper* (purple) and *Bmp4* (blue) across all 112 gene expression profiles from published studies and data generated here from whole AGM; sorted AGM endothelial, hematopoietic, and hematogenic endothelial cells (Solaimani Kartalaei et al., 2015); hematopoietic cell types (Lara-Astiaso et al., 2014); bone marrow pericytes and reticulocytes (Kunisaki et al., 2013); macrophages; E6.5–8.5 primordial germ cells and stroma (Magnúsdóttir et al., 2013); mouse embryonic fibroblasts before and after reprogramming (Pereira et al., 2013); osteoblasts (Kemp et al., 2014); and OP9 cells. The correlation coefficient is Spearman's correlation coefficient between *Bmper* and *Bmp4* across all datasets.

Tables S1–S7 are included as Excel files. Table S1 shows significant association of principal components with categorical traits. Table S2 shows genes belonging to unsupervised gene clusters 1 to 5. Table S3 shows focused hematopoiesis-related canonical pathways. Table S4 shows pathway signatures enriched in OP9 cells cultured in reaggregate versus flat cultures. Table S5 shows the common in vivo and in vitro gene expression signature underlying the HSC supportive niche. Table S6 shows p-values and adjusted p-values for differences in repopulation after treatment with recombinant proteins. Table S7 shows primer sequences of *Bmper*, *Bmp4*, and *Tbp*.

REFERENCES

Kemp, J.P., C. Medina-Gomez, K. Estrada, B. St Pourcain, D.H.M. Heppe, N.M. Warrington, L. Oei, S.M. Ring, C.J. Kruithof, N.J. Timpson, et al. 2014. Phenotypic dissection of bone mineral density reveals skeletal site specificity and facilitates the identification of novel loci in the genetic regulation of bone mass attainment. *PLoS Genet.* 10:e1004423. https://doi.org/10.1371/journal.pgen.1004423

Kunisaki, Y., I. Bruns, C. Scheiermann, J. Ahmed, S. Pinho, D. Zhang, T. Mizoguchi, Q. Wei, D. Lucas, K. Ito, et al. 2013. Arteriolar niches maintain haematopoietic stem cell quiescence. *Nature.* 502:637–643. https://doi.org/10.1038/nature12612

Lara-Astiaso, D., A. Weiner, E. Lorenzo-Vivas, I. Zaretsky, D.A. Jaitin, E. David, H. Keren-Shaul, A. Mildner, D. Winter, S. Jung, et al. 2014. Immunogenetics. Chromatin state dynamics during blood formation. *Science.* 345:943–949. https://doi.org/10.1126/science.1256271

Magnúsdóttir, E., S. Dietmann, K. Murakami, U. Günesdogan, F. Tang, S. Bao, E. Diamanti, K. Lao, B. Gottgens, and M. Azim Surani. 2013. A tripartite transcription factor network regulates primordial germ cell specification in mice. *Nat. Cell Biol.* 15:905–915. https://doi.org/10.1038/ncb2798

Pereira, C.-F., B. Chang, J. Qiu, X. Niu, D. Papatsenko, C.E. Hendry, N.R. Clark, A. Nomura-Kitabayashi, J.C. Kovacic, A. Ma'ayan, et al. 2013. Induction of a hemogenic program in mouse fibroblasts. *Cell Stem Cell.* 13:205–218. https://doi.org/10.1016/j.stem.2013.05.024

Solaimani Kartalaei, P., T. Yamada-Inagawa, C.S. Vink, E. de Pater, R. van der Linden, J. Marks-Bluth, A. van der Sloot, M. van den Hout, T. Yokomizo, M.L. van Schaick-Solernó, et al. 2015. Whole-transcriptome analysis of endothelial to hematopoietic stem cell transition reveals a requirement for Gpr56 in HSC generation. *J. Exp. Med.* 212:93–106. https://doi.org/10.1084/jem.20140767

Zakin, L., C.A. Metzinger, E.Y. Chang, C. Coffinier, and E.M. De Robertis. 2008. Development of the vertebral morphogenetic field in the mouse: interactions between Crossveinless-2 and Twisted Gastrulation. *Dev. Biol.* 323:6–18. https://doi.org/10.1016/j.ydbio.2008.08.019
